# Programmed cell death in primary Sjögren’s disease: regulatory network analysis and therapeutic prospects evaluation

**DOI:** 10.3389/fimmu.2026.1937363

**Published:** 2026-10-29

**Authors:** Wang Chengzhi, Liu Yifan, Li Songwei, Du Mengmeng, Zhu Keying, Li Huan

**Affiliations:** 1 Department of Rheumatology, The First Affiliated Hospital of Henan University of Chinese Medicine, Zhengzhou, China; 2 The First Clinical College of Henan University of Chinese Medicine, Zhengzhou, China; 3 The Second Affiliated Hospital of Henan University of Chinese Medicine, Zhengzhou, China

**Keywords:** apoptosis, autophagy, ferroptosis, primary Sjögren’s disease, pyroptosis, regulatory network, therapeutic prospects

## Abstract

Primary Sjögren’s disease (SjD) is an autoimmune disorder characterized by chronic inflammation of exocrine glands, such as the salivary and lacrimal glands. The pathogenesis of SjD remains largely unclear, and there are currently no effective curative therapies. Programmed cell death (PCD) and its regulatory network play a crucial role in the onset and progression of SjD. PCD mechanisms such as apoptosis, autophagy, pyroptosis, and ferroptosis are widely involved in the pathological mechanisms and prognosis of SjD by regulating the immune-inflammatory microenvironment, mediating the imbalance of glandular epithelial cell homeostasis, and affecting the release and clearance of self-antigens. Based on this, targeting the regulation of PCD to treat SjD has become a new therapeutic strategy. Existing evidence indicates that modulating the expression of certain miRNAs and proteins, as well as employing modern medical drugs, traditional Chinese medicine, and their active components, can influence the pathological processes of glandular inflammation and tissue damage in SjD through the regulation of apoptosis, autophagy, pyroptosis, and ferroptosis. These findings provide potential targets for precise treatment based on the PCD network.This article reviews recent relevant literature to clarify the relationship between PCD and SjD, and focuses on the molecular regulatory networks of different types of PCD in SjD and their therapeutic prospects, aiming to provide new ideas and insights for the prevention and treatment of SjD.

## 1 Introduction

Primary Sjögren’s disease (SjD) is a chronic, systemic autoimmune disease driven by autoimmune inflammation of the exocrine glands, with core manifestations including dry eyes and dry mouth. The disease primarily affects salivary and lacrimal glands, leading to progressive functional decline and even complete loss of glandular capacity, which significantly impacts patients’ quality of life (1). Currently, the precise etiology and underlying mechanisms of SjD remain incompletely understood. The scientific community generally recognizes that the disease arises from interactions among genetic susceptibility, environmental factors (e.g., viral infections), and immune dysregulation (2). Current treatment strategies for SjD mainly focus on symptomatic relief. For dryness symptoms, artificial tears, saliva substitutes, and cholinergic receptor agonists provide partial relief of glandular dysfunction. However, for systemic disease, the efficacy of available immunomodulators and biologics remains limited, and effective therapies are still lacking in clinical practice (3). Programmed cell death (PCD) is a genetically controlled, cell-autonomous, orderly process essential for various physiological and pathological processes, including embryonic development, tissue homeostasis, immune regulation, and disease pathogenesis (4). Accumulating evidence indicates that multiple forms of PCD, such as apoptosis, autophagy, pyroptosis, and ferroptosis, are closely linked to SjD pathogenesis. These pathways modulate immune cell homeostasis, glandular epithelial cell survival, and inflammatory responses, thereby collectively shaping disease progression and prognosis (5). Therefore, detailing the specific roles and regulatory networks of different PCD mechanisms in SjD pathogenesis will not only help elucidate the molecular pathology of this disease but also provide a theoretical basis for developing novel therapeutic strategies targeting cell death pathways.

## 2 Apoptosis and SjD

### 2.1 Apoptosis and its molecular mechanism

Apoptosis is a non-inflammatory form of PCD that is tightly regulated by the coordinated interplay of multiple proteins, signaling molecules, and genes, playing a central role in diverse biological processes ranging from embryonic development to tissue homeostasis (6). Apoptosis can be mainly classified into three types: the extrinsic pathway, the intrinsic pathway, and the endoplasmic reticulum stress pathway. Although the specific mechanisms of these pathways differ, they all ultimately lead to orderly apoptotic cell death (7). Among these, the intrinsic apoptotic pathway is primarily mediated by mitochondria. Upon exposure to oxidative stress, DNA damage, or endoplasmic reticulum stress, the permeability of the mitochondrial membrane changes, leading to the release of cytochrome c (Cyt c) from the mitochondria into the cytoplasm. Once released, Cyt c binds to the apoptotic protease activating factor 1 (Apaf-1), which subsequently recruits and activates caspase-9. This initiates the downstream caspase cascade and ultimately induces apoptosis (8). The extrinsic apoptotic pathway is mainly death receptor-mediated, involving TNFR1 and Fas. Ligand binding induces receptor trimerization and adaptor protein recruitment, leading to the formation of the death-inducing signaling complex (DISC). DISC then activates initiator caspase-8, which in turn triggers the downstream apoptotic cascade (9). The endoplasmic reticulum stress pathway is a key signaling pathway that is activated when endoplasmic reticulum (ER) homeostasis is disrupted. Under normal circumstances, glucose-regulated protein 78 (GRP78) binds to three endoplasmic reticulum membrane proteins, PERK, IRE1, and ATF6, and remains inactive; when the endoplasmic reticulum homeostasis is disrupted, GRP78 dissociates from these proteins, thereby initiating the unfolded protein response. If the stress persists, the UPR upregulates the expression of the pro-apoptotic factor CHOP through three main branches: the PERK-eIF2α-ATF4, IRE1-XBP1-JNK, and ATF6 pathways. CHOP can downregulate the expression of anti-apoptotic protein Bcl-2, upregulate the expression of pro-apoptotic protein Bim, and activate caspase-12 and its downstream caspase-3, ultimately inducing apoptosis (10).

### 2.2 Relationship between apoptosis and SjD

In the early stage of SjD, exocrine gland cells undergo apoptosis. Epithelial cell apoptosis not only leads to the production of anti-SSA and anti-SSB antibodies but also promotes the infiltration of autoreactive lymphocytes, thereby accelerating disease progression (11). As the disease progresses, sustained apoptosis of salivary gland epithelial cells (SGECs) further triggers autoimmunity and impairs glandular function (12). Furthermore, in the labial glands (LG) of SjD patients, the release of mitochondrial Cyt c into the cytoplasm is significantly increased, and its expression level is positively correlated with disease severity. This finding suggests that activation of the mitochondrial apoptotic pathway plays a critical role in the pathogenesis of SjD (13).

### 2.3 Therapeutic strategies targeting apoptosis

Apoptosis is the core mechanism underlying salivary gland (SG) damage in SjD. Inhibiting abnormal apoptosis in SjD therefore represents a highly promising therapeutic strategy. As key regulators of apoptosis, miRNAs profoundly influence disease progression and therapeutic outcomes in SjD. Current studies have shown that different miRNAs can affect the onset and progression of SjD by either promoting or inhibiting apoptosis. Specifically, upregulation of miR-155-5p induces apoptosis and promotes SjD progression, whereas downregulation of miR-155-5p inhibits apoptosis and alleviates disease progression. Furthermore, downregulation of miR-34a or miR-223-3p, as well as upregulation of miR-1207-5p/miR-4695-3p or miR-23b-3p, inhibits apoptosis and holds therapeutic potential for SjD. (The mechanism of the effect of regulating apoptosis on SjD is shown in **Figure 1**).

**Figure 1:**
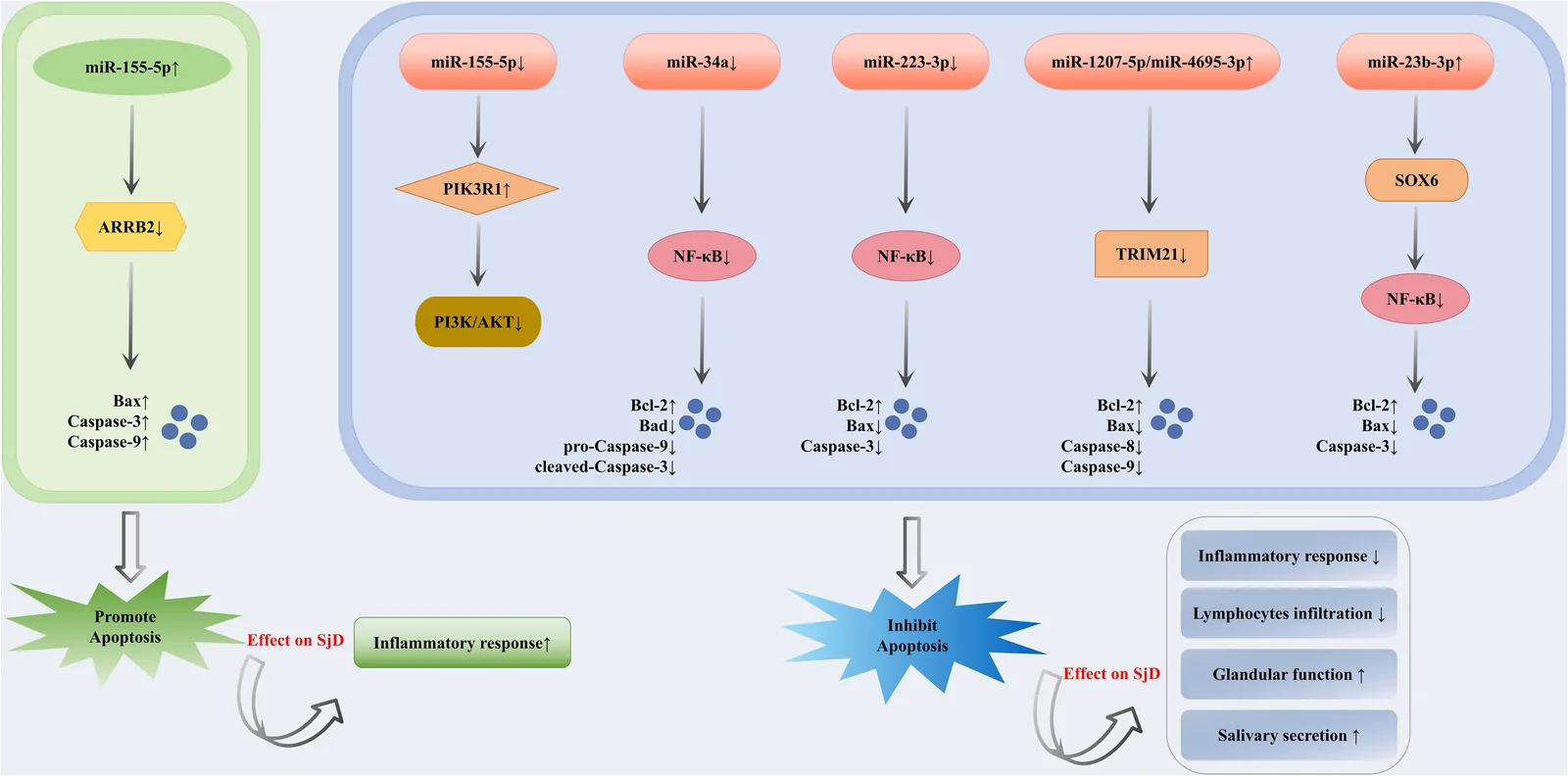
The mechanism of the effect of regulating apoptosis on SjD.

miR-155-5p plays a crucial role in regulating the differentiation and development of T and B lymphocytes (14), and its expression is significantly increased in peripheral blood mononuclear cells of patients with SjD (15). Upregulation of miR-155-5p targets and downregulates β-arrestin 2 (ARRB2) expression, leading to an increased p-I-κB/I-κB ratio and elevated p65 expression. This activates the NF-κB pathway, upregulates Bax, Caspase-3, and Caspase-9, and induces apoptosis of SGECs. Concurrently, it increases the expression of TNF-α and IL-6, thereby exacerbating the inflammatory response (16). It should be noted that this conclusion is based on the immortalized SGECs cell line, which has limited physiological relevance and requires validation using primary cells or *in vivo* models. The regulatory subunit of phosphatidylinositol-3-kinase (PIK3R1) negatively regulates the PI3K/AKT pathway by binding to the catalytic subunit p110. Consequently, inhibition of PIK3R1 leads to upregulated phosphorylation of PI3K and AKT (17). Downregulation of miR-155-5p targets and upregulates PIK3R1 expression, thereby reducing the p-PI3K/PI3K and p-AKT/AKT ratios, inhibiting the PI3K/AKT pathway, and suppressing apoptosis in human salivary gland epithelial cells (HSGECs). Additionally, this increases IL-4 and IL-10 expression, decreases TNF-α and IL-6 expression, alleviates the inflammatory response, improves glandular function, and increases salivary secretion in non-obese diabetic (NOD) mice (18). Among them, the cell-level conclusion is based on immortalized HSGECs and has limited physiological relevance; the results of NOD mice are closer to the *in vivo* environment, but species differences still limit their direct transformation to clinical applications. Therefore, miR-155-5p can regulate cell apoptosis and inflammatory responses in both directions. Developing its inhibitor is expected to become an effective strategy for treating SjD, but its clinical transformation still requires systematic verification through human organoids, humanized mice, and clinical trials. miR-34a expression is elevated in the tear fluid of patients with SjD (19). Downregulating miR-34a reduces NF-κB p65, increases IκBα, inhibits the NF-κB pathway, and upregulates Bcl-2. It also downregulates Bad, pro-Caspase-9, Caspase-9, cleaved Caspase-3, and Cyt c, thereby inhibiting apoptosis of HSGECs and ultimately alleviating glandular damage and increasing salivary secretion in NOD mice (20). The cell-level conclusions of this study are based on immortalized HSGE cell lines and have limited physiological relevance; although the results in NOD mice are closer to the *in vivo* environment, species differences still limit their direct application in clinical settings. The expression of miR-223-3p in peripheral blood mononuclear cells (PBMCs) of SjD patients was significantly increased (21), this suggests a connection with glandular damage; however, this observation is based on patient tissue samples, and the sample size and population heterogeneity still need to be verified in a larger cohort. Downregulating miR-223-3p inhibits the NF-κB pathway, upregulates Bcl-2, and downregulates Bax and Caspase-3, thereby inhibiting SGECs apoptosis. Additionally, it reduces TNF-α, IL-6, and IL-12 expression, alleviates the inflammatory response, and increases tear secretion (22). However, the cellular-level conclusions of this study are based on immortalized SGECs cell lines, which have limited physiological relevance; the data on improved tear secretion come from the NOD mouse model, which is closer to the *in vivo* environment, but species differences still limit its direct application in clinical practice. Anti-Ro/SSA and anti-La/SSB antibodies are characteristic serological markers of SjD. Among these, tripartite motif-containing protein 21 (TRIM21), the specific antigen targeted by anti-Ro/SSA antibodies, plays a crucial role in regulating apoptosis (23). miR-1207-5p and miR-4695-3p are significantly downregulated in the SG tissues of SjD patients, this suggests that it may be related to glandular damage; however, this observation is based on patient tissue samples, and the sample size and population heterogeneity still need to be verified in a larger cohort. Upregulation of miR-1207-5p and miR-4695-3p targets and downregulates TRIM21, increases Bcl-2, reduces Bax, Caspase-8, and Caspase-9, and inhibits apoptosis of HSGECs (24). It should be noted that this conclusion is based on immortalized HSG cell lines, and its physiological relevance is limited. Further validation using primary cells or *in vivo* models is required. MiR-23b-3p exhibits significant anti-apoptotic activity (25). Upregulation of miR-23b-3p targets SRY-related HMG box transcription factor 6 (SOX6), thereby inhibiting the NF-κB pathway. This leads to increased Bcl-2 and decreased Bax and Caspase-3, ultimately suppressing apoptosis of submandibular gland (SMG) cells. Additionally, miR-23b-3p upregulation reduces TNF-α, IL-6, and IL-17A expression, alleviates inflammatory responses, lowers SSA/Ro and SSB/La antibody levels, inhibits lymphocyte infiltration in NOD mice, and increases salivary secretion (26). However, the conclusions at the cellular level are based on immortalized SMG cell lines, which have limited physiological relevance; the results from NOD mice are closer to the *in vivo* environment, but species differences still limit their direct application in clinical settings.

In addition to miRNAs, various other molecules and cytokines can bidirectionally influence SjD progression by regulating apoptosis. On one hand, upregulation of B7-H3, CTRP3 can induce apoptosis and promote disease progression. On the other hand, downregulation of β-arrestin2 or JNK, as well as treatment with SHED-exos, ME-MSCs, IL-11, or alemtuzumab, can inhibit apoptosis and exert therapeutic effects.

B7-H3 expression is upregulated in the SG tissues of SjD patients, this suggests a connection with glandular damage, but this observation is based on patient tissue samples. The sample size and population heterogeneity still need to be verified in a larger cohort. Overexpressed B7-H3 increases p65, activates the NF-κB pathway, upregulates Bax and Caspase-3, and downregulates Bcl-2, thereby promoting submandibular gland cells (HSGE) apoptosis. Additionally, it increases TNF-α and IL-6 expression, exacerbating the inflammatory response, and reduces AQP5 expression, leading to glandular dysfunction (27). It is worth emphasizing that this discovery is based on immortalized HSGE cells, which have limited physiological relevance and still requires validation using primary cells or *in vivo* models. C1q/tumor necrosis factor-related protein 3 (CTRP3) level is significantly elevated in the serum and SMG of SjD patients and NOD mice. SMG-C6 cells are an immortalized cell line derived from rat SMG. CTRP3 upregulates TNFR1 and inhibits c-FLIP, thereby promoting the formation of the FADD/RIPK1/Caspase-8 complex II and activating the TNFR1-mediated death pathway. Consequently, it upregulates Bax, Caspase-3, Caspase-8, Caspase-10, cleaved Caspase-3, and Cyt c, while downregulating Bcl-2. These changes promote SMG-C6 cell apoptosis, disrupt the SGECs barrier, and lead to functional impairment (28). It should be noted that this conclusion is based on the immortalized SMG-C6 cell line, and its physiological relevance is limited. Further validation using primary cells or *in vivo* models is required. In the LG of SjD patients, the GRP78/activating transcription factor 6 (ATF6) signaling pathway is overactivated and participates in apoptosis by activating CHOP (29). This suggests that this pathway may be related to glandular damage; however, this observation is based on patient tissue samples, and the sample size and population heterogeneity still need to be verified in a larger cohort. Downregulating β-arrestin2 inhibits the GRP78/ATF6/CHOP pathway, prevents HSGE apoptosis, reduces lymphocyte infiltration in the glandular tissue of NOD mice, improves glandular function, and enhances salivary secretion (30). However, the conclusions at the cellular level are based on immortalized HSGECs cell lines, which have limited physiological relevance; although the results in NOD mice are closer to the *in vivo* environment, species differences still limit their direct application in clinical settings. Human-derived keratinocyte-free dental pulp stem cell exosomes (SHED-exos) downregulate RAS, MEK1/2, and p-ERK1/2, inhibit the RAS/MEK/ERK pathway, reduce cleaved Caspase-3, and suppress SMG-C6 cell apoptosis. Additionally, they alleviate lymphocyte infiltration in the SMG and restore salivary secretion function in NOD mice (31). Among them, the cell-level conclusions were based on the rat-derived immortalized SMG-C6 cell line, and there were species differences; although the results of NOD mice were closer to the *in vivo* environment, species differences still limited their direct application in clinical settings. Furthermore, this study also found that SHED-exos upregulate GPX4, downregulate ACSL4, and inhibit ferroptosis in SMG-C6 cells; downregulate KEAP1 and inhibit oxidative stress-induced cell death; and downregulate RIPK3 and inhibit necroptosis. Melatonin (MLT) is a naturally occurring indoleamine substance that exhibits multiple biological and pharmacological effects, including anti-inflammatory, antioxidant, and anti-apoptotic properties (32). MLT downregulates STAT3 and p-STAT3, inhibits the IL-6/STAT3 pathway, increases Bcl-2, and reduces Bax, cleaved caspase-3, and Cyt c, thereby inhibiting HSGE apoptosis. It also increases TGF-β and IL-10, reduces IFN-γ, TNF-α, IL-6, IL-17A, and IL-21, and alleviates the inflammatory response. Moreover, MLT upregulates NRF2, NQO1, SOD, and GSH, while downregulating ROS and MDA, thereby inhibiting oxidative stress and reducing glandular damage in NOD mice. Furthermore, it increases AQP5 expression, improves glandular function, and enhances salivary secretion (33). The cell-level conclusions of this study are based on immortalized HSGE cell lines and have limited physiological relevance; although the results in NOD mice are closer to the *in vivo* environment, species differences still limit their direct application in clinical settings. Iguratimod (IGU) is a commonly used immunosuppressant. It can effectively reduce erythrocyte sedimentation rate (ESR), IgG levels, and platelet count in patients with SjD, and alleviate symptoms such as dry mouth and dry eyes (34). IGU downregulates Bax and Caspase-3, inhibits apoptosis of SMG epithelial cells; reduces TNF-α, IL-1β, IL-6, and IL-17, alleviating the inflammatory response; reduces lymphocyte infiltration in the SMG of NOD mice, and increases salivary secretion (35). However, the cellular-level conclusions of this study are based on SMG epithelial cells, and their physiological relevance is limited by the constraints of the cell model; although the results of NOD mice are closer to the *in vivo* environment, species differences still restrict their direct application in clinical settings. Mesenchymal stem cells (MSCs) exhibit immunomodulatory functions and multi-directional differentiation characteristics under specific culture conditions (36). Mouse embryonic MSCs (ME-MSCs) can inhibit apoptosis of SGECs and promote their proliferation, thereby protecting the SG structure and function in NOD mice and increasing salivary secretion (37). Among them, the cell-level conclusions are based on immortalized SGECs cell lines, which have limited physiological relevance; ME-MSCs are derived from mouse embryos and have species differences from human MSCs; although the results of NOD mice are closer to the *in vivo* environment, species differences still limit their direct transformation for clinical use. The expression of IL-11 in the LG of SjD patients is significantly downregulated and is significantly positively correlated with C-reactive protein (CRP) levels. Further mechanistic studies have revealed that IL-11 inhibits HSG apoptosis, promotes their proliferation and survival, improves glandular function, and increases salivary secretion (38). It should be noted that the conclusion of this mechanism is based on immortalized HSG cell lines, and its physiological relevance is limited. Further validation using primary cells or *in vivo* models is required. In the SGECs of SjD patients, abnormal expression and activation of Toll-like receptors (TLRs) can trigger an inflammatory response (39). TLR9 is overexpressed in the epithelial cells of the lacrimal gland (LG) ducts and acinar cells of SjD patients (40). Upregulation of TLR9 expression promotes HSG apoptosis and reduces the survival rate of these cells; conversely, inhibiting the JNK signaling pathway downregulates the Bax/Bcl-2 ratio and reduces Caspase-3 activity, thereby inhibiting HSG apoptosis (41). It should be noted that the conclusion of this mechanism is based on immortalized HSG cell lines, and its physiological relevance is limited. Further validation using primary cells or *in vivo* models is required. Therefore, the therapeutic potential of developing JNK-targeted inhibitors for SjD remains a clinical preclinical hypothesis at present, and its translational feasibility needs to be further evaluated.

In addition, Chinese herbal medicine (such as AS-OD), active components of Traditional Chinese medicine (TCM) (such as *Dendrobium officinale* polysaccharide, Epigallocatechin gallate, and Quercetin), and TCM compound formulas such as Zengye Decoction can exert anti-SjD effects by inhibiting apoptosis, reducing inflammatory responses, restoring the Th17/Treg immune balance, and suppressing oxidative stress. (The mechanism of the effect of regulating apoptosis on SjD is shown in **Figure 2**).

**Figure 2:**
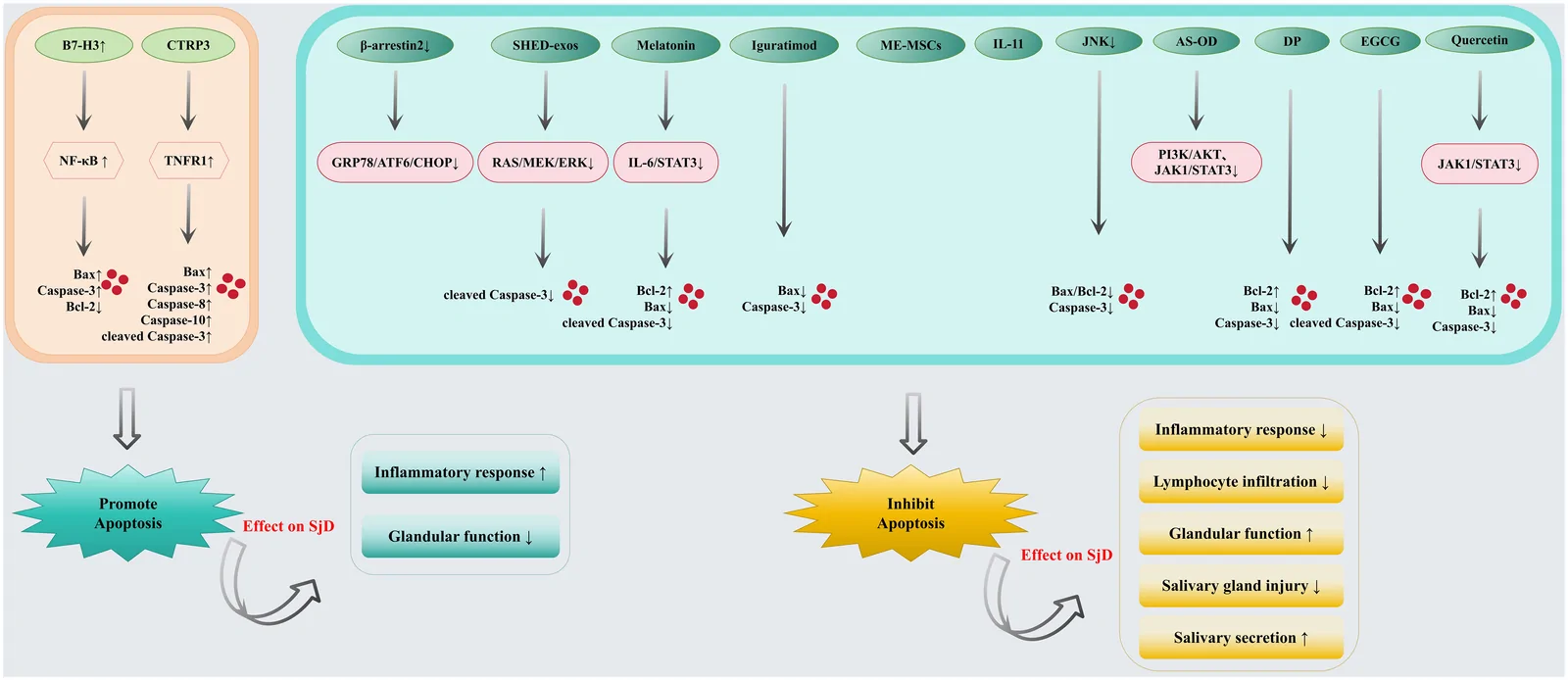
The mechanism of the effect of regulating apoptosis on SjD.

Astragalus (42), Ophiopogon (43) and Dendrobium (44), and others all exhibit significant anti-SjD effects. Meanwhile, the herbal combination consisting of Astragalus-Salvia (AS) and Ophiopogon-Dendrobium (OD) (AS-OD) is a commonly used clinical treatment for SjD. The A253 cell is a cell line derived from SG. AS-OD inhibits the PI3K/AKT and JAK1/STAT3 pathways, thereby reducing A253 cell apoptosis and promoting their survival. Additionally, it reduces TNF-α, IL-1β, and IL-6 levels, alleviates the inflammatory response, restores the Th17/Treg immune balance, and downregulates anti-SSA/Ro and anti-SSB/La antibodies. Furthermore, AS-OD upregulates AQP5 expression, which improves glandular function, reduces lymphocyte infiltration, and increases salivary secretion in NOD mice (45). Among them, the cell-level conclusion is based on the immortalized A253 cell line, and its physiological relevance is limited; although the results of NOD mice are closer to the *in vivo* environment, species differences still restrict its direct transformation for clinical use. *Dendrobium officinale* polysaccharide (DP) upregulates AQP5 expression, improves glandular function, and increases salivary secretion (46). In addition, DP upregulates Bcl-2, downregulates Bax and Caspase-3, and inhibits SG cell apoptosis. It also reduces TNF-α, IL-1β, and IL-6, alleviates the inflammatory response, and improves glandular function and increases salivary secretion in NOD mice (47). Among them, the cell-level conclusion is based on the SG cell line and has limited physiological relevance; the results from NOD mice are closer to the *in vivo* environment, but species differences still limit their direct application in clinical settings. The MRL/lpr mouse is a classic spontaneous model for SjD-associated autoimmune sialadenitis and serves as a valuable addition or alternative to other SjD mouse models (48). Epigallocatechin gallate (EGCG) upregulates Bcl-2 and downregulates Bax, p53, and cleaved Caspase-3, thereby inhibiting SGECs apoptosis. It also inhibits reactive oxygen species (ROS)-mediated DNA damage and alleviates oxidative stress, leading to improved SG function and increased salivary secretion in MRL/lpr mice, as well as reduced serum levels of SSA/Ro and SSB/La autoantibodies (49). Among them, the cell-level conclusion is based on immortalized SGECs cell lines and has limited physiological relevance; the results from MRL/lpr mice, although derived from the classic SjD model, are still limited in direct clinical application due to species differences and strain specificity. Quercetin (QUE) is a natural flavonoid compound with antioxidant, anti-inflammatory and anti-apoptotic activities (50). QUE can reduce the expression of AQP5 and M3R, alleviate the SG damage in NOD mice, and increase salivary secretion (51). A study has shown that QUE, by targeting and inhibiting the LP/OB-R-mediated JAK2/STAT3 signaling pathway, upregulates Bcl-2 and downregulates Bax, Caspase-3, and cleaved Caspase-3, thereby inhibiting SGECs apoptosis. Additionally, it reduces TNF-α and IL-6, alleviates the inflammatory response and lymphocyte infiltration, improves glandular damage, and increases salivary secretion in NOD mice (52). Among them, the cell-level conclusion is based on immortalized SGECs cell lines and has limited physiological relevance; the results from NOD mice are closer to the *in vivo* environment, but species differences still limit their direct transformation for clinical use. Zengye Decoction (ZYD) is a classic prescription for treating SjD. A study has shown that ZYD can significantly improve the structure and function of the SMG in NOD mice (53). In addition, ZYD can upregulate Bcl-2, downregulate Bax and Caspase-3, and inhibit SMG cell apoptosis. It can also reduce TNF-α, IL-1β, IL-6, and IL-17, alleviate the inflammatory response and lymphocyte infiltration, and increase salivary secretion (54). Among them, the cell-level conclusions are based on immortalized SMG cell lines and have limited physiological relevance; the data on salivary secretion improvement mostly come from NOD mouse models, although they are closer to the *in vivo* environment, species differences still limit their direct application in clinical practice. (The mechanism of the effect of regulating apoptosis on SjD is summarized in **Table 1**).

**Table 1:** Summary of the mechanisms of the effect of regulating apoptosis on SjD.

Intervention	Model	Signaling pathway	Apoptosis regulation	Molecular mechanism	Synergistic mechanism	Effect on SjD	References
miR-155-5p↑	SGECs	NF-κB ↑	Promote	Bax, Caspase-3, Caspase-9 ↑	Inflammatory response ↑	Drive SjD progression	(16)
miR-155-5p↓	HSGECs, NOD mice	PI3K/AKT ↓	Inhibit	unspecified	Inflammatory response ↓, glandular function ↑	Suppress SjD progression	(18)
miR-34a↓	HSGE, NOD mice	NF-κB ↓	Inhibit	Bcl-2 ↑, Bad, pro-Caspase-9, Caspase-9, cleaved Caspase-3, Cyt c ↓	Salivary gland injury ↓	Suppress SjD progression	(20)
miR-223-3p↓	SjD patients, SGECs	NF-κB ↓	Inhibit	Bcl-2 ↑, Bax, Caspase-3 ↓	Inflammatory response ↓	Suppress SjD progression	(22)
miR-1207-5p/miR-4695-3p↑	SjD patients, HSG	unspecified	Inhibit	Bcl-2 ↑, Bax, Caspase-8, Caspase-9 ↓	unspecified	Suppress SjD progression	(24)
miR-23b-3p↑	SMG cells, NOD mice	NF-κB ↓	Inhibit	Bcl-2 ↑, Bax, Caspase-3 ↓	Inflammatory response ↓, lymphocytes infiltration ↓	Suppress SjD progression	(26)
B7-H3↑	SjD patients, HSGE, NOD mice	NF-κB ↑	Promote	Bax, Caspase-3 ↑, Bcl-2 ↓	Inflammatory response ↑, glandular function ↓	Drive SjD progression	(27)
CTRP3	SjD patients, NOD mice, SMG-C6 cells	TNFR1 ↑	Promote	Bax, Caspase-3/8/10, cleaved Caspase-3, Cyt c ↑, Bcl-2 ↓	Glandular function ↓	Drive SjD progression	(28)
β-arrestin2↓	HSGECs, ESS mice	GRP78/ATF6/CHOP ↓	Inhibit	unspecified	Gland function ↑, lymphocyte infiltration ↓	Suppress SjD progression	(30)
SHED-exos	SMG-C6 cells, NOD mice	RAS/MEK/ERK ↓	Inhibit	cleaved Caspase-3 ↓	Ferroptosis, oxidative death, necroptosis ↓, landular function ↑, lymphocyte infiltration ↓	Suppress SjD progression	(31)
Melatonin	HSG, NOD mice	IL-6/STAT3 ↓	Inhibit	Bcl-2 ↑, Bax, cleaved Caspase-3, Cyt c ↓	Inflammatory response ↓, glandular function ↑	Suppress SjD progression	(33)
Iguratimod	SMG epithelial cells, NOD mice	unspecified	Inhibit	Bax, Caspase-3 ↓	Inflammatory response ↓, lymphocyte infiltration ↓	Suppress SjD progression	(35)
ME-MSCs	SGECs, NOD mice	unspecified	Inhibit	unspecified	Glandular function ↑	Suppress SjD progression	(37)
IL-11	SjD patients, HSG	unspecified	Inhibit	unspecified	Glandular function ↑	Suppress SjD progression	(38)
JNK↓	HSG	unspecified	Inhibit	Bax/Bcl-2, Caspase-3 ↓	unspecified	Suppress SjD progression	(41)
AS-OD	A253 Cells	PI3K/AKT, JAK1/STAT3 ↓	Inhibit	unspecified	Inflammatory response ↓, Restore the Th17/Treg immune balance, lymphocyte infiltration ↓, Glandular function ↑	Suppress SjD progression	(45)
*Dendrobium officinale* polysaccharide	SG cells, NOD mice	unspecified	Inhibit	Bcl-2 ↑, Bax, Caspase-3 ↓	Inflammatory response ↓, Glandular function ↑	Suppress SjD progression	(47)
Epigallocatechin gallate	SGECs, MRL/lpr mice	unspecified	Inhibit	Bcl-2 ↑, Bax, cleaved Caspase-3 ↓	Inflammatory response ↓, Glandular function ↑	Suppress SjD progression	(49)
Quercetin	SGECs, NOD mice	JAK2/STAT3 ↓	Inhibit	Bcl-2 ↑, Bax, Caspase-3, cleaved Caspase-3 ↓	Inflammatory response ↓, lymphocyte infiltration ↓, Glandular function ↑	Suppress SjD progression	(52)
Zengye Decoction	SMG cells, NOD mice	unspecified	Inhibit	Bcl-2 ↑, Bax, Caspase-3 ↓	Inflammatory response ↓, lymphocyte infiltration ↓	Suppress SjD progression	(54)

↑, Increase/Activate/Enhance; ↓, Decrease/Lower/Inhibit.

(“Unspecified” indicates that the original literature only reported the regulation of PCD effect molecules or inflammatory factors by the intervention measures, but did not further clarify the upstream signaling pathways. Such studies mainly focus on phenotypic validation and have not yet conducted mechanism analysis at the pathway level. This suggests that subsequent research needs to supplement pathway validation experiments. It should be noted that “Unspecified” does not mean that the intervention measure does not have signal pathway regulation, but reflects the current insufficient depth of mechanism analysis in the research).

## 3 Autophagy and SjD

### 3.1 Autophagy and its molecular mechanism

Autophagy is a highly conserved cellular catabolic process that primarily degrades damaged organelles and misfolded proteins via the lysosomal pathway, thereby maintaining cellular homeostasis and promoting material recycling (55). The core process of autophagy involves the formation, transportation, and fusion of autophagosomes. Under normal physiological conditions, autophagy-related genes (ATGs) work together to initiate the autophagic process. First, in response to stress signals such as nutrient deprivation and reactive oxygen species, the UNC-51-like kinase 1/2 (ULK1/2) kinase complex is activated, mediating autophagosome nucleation. Subsequently, the autophagy-related protein microtubule-associated protein light chain 3 (LC3) is converted from LC3-I to LC3-II and anchors to the autophagosome membrane, participating in autophagosome elongation and maturation. The autophagosome gradually encapsulates damaged organelles, protein aggregates, and other substrates, eventually forming a complete autophagosome. Finally, the autophagosome fuses with the lysosome, and the hydrolytic enzymes within the lysosome degrade the substrates, enabling the recycling and reuse of cellular materials (56–58).

### 3.2 Relationship between autophagy and SjD

Abnormal autophagy of immune cells, particularly T lymphocytes and B lymphocytes, is closely associated with the pathological damage and disease progression of SjD. The main pathological mechanism of SjD is the infiltration of T lymphocytes in the exocrine glands, with CD4^+^ T cells playing a dominant role in inducing glandular lesions. Autophagy activity in CD4^+^ T cells is significantly upregulated in the serum of SjD patients (59). Autophagy activation in T lymphocytes may represent the primary pathological change in the early stage of SjD and is directly correlated with disease activity (60). Autophagy is a common feature of infiltrating T cells and B cells in the minor salivary glands (MSG) of patients with SjD. Aberrant regulation of autophagy has been observed in germinal center-like structures within the MSG, suggesting that autophagy may be involved in the development and progression of the local autoimmune process in SjD (61). The CXC motif chemokine receptor 5 (CXCR5) plays a crucial role in promoting the autophagic response. CXCR5 expression is significantly increased in the LG of SjD patients (62). High expression of LC3-II has been observed in the SGECs of SjD patients, and increased autophagic activity is positively correlated with glandular damage (63). The levels of the autophagy markers ATG5 and LC3-II in the tear film and conjunctival epithelium of patients with SjD-related dry eye (SjD-DE) are significantly elevated and correlate with disease severity. Local corticosteroid treatment reduces these levels, suggesting that these autophagy markers have the potential to serve as diagnostic and therapeutic monitoring biomarkers for SjD-DE (64).

### 3.3 Therapeutic prospects of targeted autophagy

In SjD, autophagy exerts a bidirectional regulatory effect on disease progression, acting as a double-edged sword. Comprehensive literature analysis indicates that autophagy can be either activated or inhibited by different regulatory factors, thereby producing opposite functional effects. On one hand, H3K18la, U0126/Rapamycin, and S1P can promote autophagy. Among these, H3K18la can accelerate the course of SjD, whereas U0126/Rapamycin, and S1P exert anti-SjD effects. On the other hand, SOD3, chloroquine, artesunate combined with metformin, and blueberry leaf extract can all inhibit autophagy, thereby ameliorating the dry mouth and dry eye symptoms of SjD. These opposing regulatory factors suggest that the functional orientation of autophagy is not fixed, and its bidirectionality can be comprehensively understood from the following two aspects. The first is the heterogeneity of target cell types. In SGECs, autophagy has been proven to be a survival mechanism, its activation is negatively correlated with apoptosis, and it promotes the expression of adhesion molecules and cell activation, while inhibiting autophagy can weaken epithelial cell activation; in infiltrating lymphocytes, the expression of autophagy-related genes increases with the severity of inflammatory infiltration, and this is particularly significant in B cells, suggesting that autophagy may support the survival and activation of autoreactive lymphocytes. The same pathway shows opposite pathological significance in different cell types, providing a cellular-level explanation for the “double-edged sword” phenomenon. The second is disease stage dependence. The epidemiological characteristics of SjD patients and the association between age-related macrophage autophagy impairment and M1 polarization suggest that the function of autophagy may evolve with the disease process; some scholars have proposed that the temporal relationship between autophagy and apoptosis in SjD may have undergone a transformation from “autophagy compensatory protection” to “apoptosis initiation after autophagy exhaustion”, but this model lacks direct verification with longitudinal temporal data. Overall, the evidence supporting the two explanations shows significant asymmetry. The heterogeneity of cell types has been supported by *in vitro* cell experiments and immunohistochemical studies, while stage dependence is still mainly based on cross-sectional observations and theoretical deductions. Its verification requires longitudinal comparisons of samples from different disease stages. This evidence pattern suggests that future research should prioritize attention to cell type specificity when evaluating the role of autophagy in SjD, and consider disease stage as a variable that needs further verification, rather than an established premise. (The mechanism of the effect of regulating autophagy on SjD is shown in **Figure 3**).

**Figure 3:**
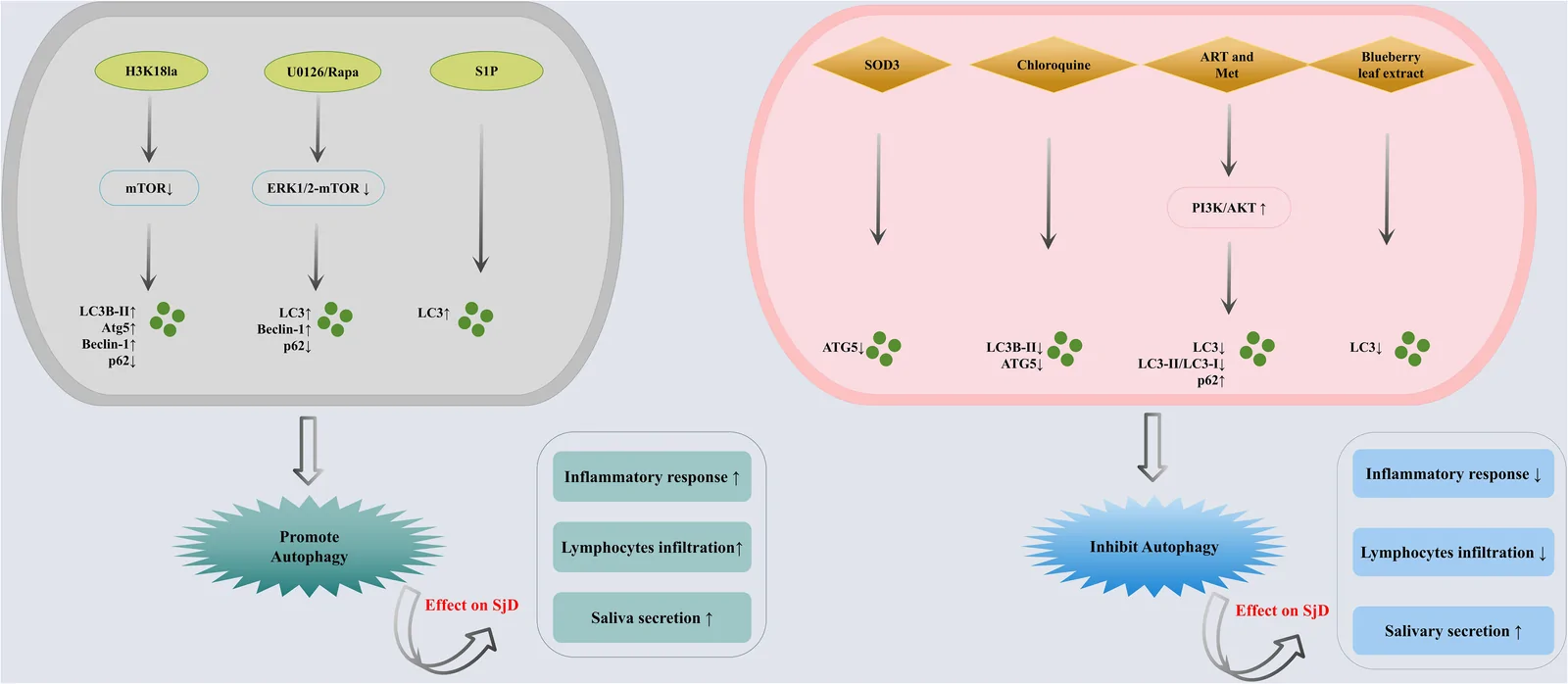
The mechanism of the effect of regulating autophagy on SjD.

A previous study has suggested that lactic acid may play a significant role in the pathogenesis of SjD (65). In patients with SjD, lactate accumulation in the serum and SG is increased. Lactate enters SG epithelial cells via lactate transporters and induces histone H3K18 lactylation (H3K18la). H3K18la inhibits mTOR activity, upregulates LC3B-II, Atg5, and Beclin-1 expression, and downregulates p62 expression, thereby inducing autophagy in A253 cells. Concurrently, H3K18la aggravates the inflammatory response in the SG of NOD mice, increases lymphocyte infiltration, and disrupts glandular function (66). Among them, the cell-level conclusion is based on the immortalized A253 cell line, and its physiological relevance is limited; although the results of NOD mice are closer to the *in vivo* environment, species differences still restrict its direct transformation for clinical use. Anti-Ro52/SSA autoantibodies are closely associated with early onset and glandular dysfunction in SjD, and a decrease in their levels reflects improved glandular function (67). Thus, targeting the inhibition of anti-Ro52/SSA autoantibody production or expression is a key therapeutic strategy for SjD. In SjD, increased endoplasmic reticulum stress activates the ERK1/2-mTOR signaling pathway, thereby inhibiting autophagy. This leads to abnormally high expression of Ro52/SSA self-antigens in the LG tissues of SjD patients, the mandibular gland tissues of NOD mice, and HSGECs, as well as their release into the extracellular space. MEK1/2 inhibitors (U0126) or Rapamycin (Rapa) inhibit the ERK1/2-mTOR pathway, upregulate LC3 and Beclin-1 expression, downregulate p62 expression, thereby promoting autophagy, reducing Ro52/SSA expression, and inhibiting its extracellular transfer (68). Among them, the patient tissue evidence suggests that this mechanism is related to human SjD, but the sample size and heterogeneity still need to be verified; the results of NOD mice are closer to the *in vivo* environment, but species differences still limit their direct application to clinical practice; the conclusions at the cellular level are based on immortalized HSGECs and have limited physiological relevance. In addition, the target organ delivery efficiency of U0126 and Rapa and their safe doses in SjD patients still need further research. Sphingosine-1-phosphate (S1P), a bioactive sphingolipid metabolite, holds significant research value in SjD. S1P upregulates LC3 expression and promotes autophagy in regulatory T cells (Tregs), thereby restoring their function and increasing their numbers. Concurrently, S1P enhances Treg proliferation by upregulating Ki67 expression. It also reduces IFN-γ levels while upregulating IL-10 and IL-13, collectively alleviating the inflammatory response. In the NOD mouse model of SjD, S1P treatment reduces glandular lymphocytic infiltration, lowers serum levels of anti-SSA/Ro and anti-SSB/La autoantibodies, decreases organ indices, and improves salivary secretion (69). Among them, the conclusions related to Tregs are based on primary immune cells cultured *in vitro*, which are closer to the physiological state than immortalized cell lines. However, the *in vitro* environment still cannot fully reflect the immune microenvironment *in vivo*. The results of NOD mice are closer to the *in vivo* environment, but species differences still limit their direct transformation for clinical application. Superoxide dismutase (SOD) is a key antioxidant enzyme that can neutralize superoxide free radicals. It is regarded as a potential target for the treatment of SjD-DE (70). Superoxide dismutase 3 (SOD3) down-regulates ATG5 expression and inhibits autophagy in the LG. It also reduces CD3 and B220 levels, thereby suppressing the infiltration of T and B lymphocytes. Furthermore, SOD3 decreases the expression of TNF-α, IL-1β, and IL-6, alleviates the inflammatory response, and ameliorates conjunctival histological damage in NOD mice. Consequently, it lowers the corneal staining score and increases tear secretion (71). It should be noted that the above conclusion is mainly based on the NOD mouse model, which is closer to the *in vivo* environment, but species differences still limit its direct application in clinical practice; in addition, the delivery efficiency of SOD3 to target organs and the safe dosage in SjD-DE patients still require further research. Chloroquine (CQ) is an antimalarial drug that has immunomodulatory effects and can alleviate the dry eye symptoms and extra-ocular manifestations of SjD (72). In the early stage of SjD, CQ down-regulates the expression of LC3B-II and ATG5, thereby inhibiting autophagy. It also reduces the levels of TNF-α, IL-1β, and IL-6, as well as the level of anti-Ro antibodies, consequently alleviating the inflammatory response and tear gland damage. Additionally, CQ decreases the expression of B220 and CD3, thereby reducing the infiltration of T and B lymphocytes in the tear gland. Ultimately, these effects lead to a lower corneal staining score and increased tear secretion in NOD mice (73). The above mechanism conclusions are mainly based on the NOD mouse model, which is closer to the *in vivo* environment, but species differences still limit its direct application in clinical practice. In addition, the target organ delivery efficiency of CQ and its long-term safety dosage in SjD patients still require further research. Artesunate (ART) is a water-soluble derivative of artemisinin, featuring low toxicity, good tolerance and extensive pharmacological activities (74). Metformin (Met) is a synthetic biguanide drug. Currently, it is the first-line medication used in clinical settings for the treatment and prevention of type 2 diabetes (T2DM) and its various complications (75). The combined use of ART and Met upregulates the p-AKT/AKT and p-PI3K/PI3K ratios, thereby activating the PI3K/AKT pathway. This treatment also reduces LC3 expression and the LC3-II/LC3-I ratio while upregulating p62 expression, collectively indicating the inhibition of autophagy. Furthermore, the combination therapy increases AQP5 expression, alleviates pathological damage to the SMG, improves glandular function in NOD mice, and enhances salivary secretion (76). The above conclusion is mainly based on the NOD mouse model, which is closer to the *in vivo* environment, but species differences still limit its direct application to clinical practice. In addition, the optimal ratio of ART combined with Met, the efficiency of target organ delivery, and the safety in SjD patients still require further research. Blueberry leaf extract (BLEx) downregulates LC3 expression, inhibits autophagy in the LG; reduces TNF-α and IL-1β expression, alleviates the inflammatory response, and increases tear secretion in NOD mice (77). The above conclusion is mainly based on the NOD mouse model, which is closer to the *in vivo* environment, but species differences still limit its direct application in clinical practice. In addition, further research is needed on the active ingredients of BLEx, the efficiency of target organ delivery, and the safe dosage in patients with SjD. (The mechanism of the effect of regulating autophagy on SjD is summarized in **Table 2**).

**Table 2:** Summary of the mechanisms of the effect of regulating autophagy on SjD.

Intervention	Model	Signaling pathway	Autophagy regulation	Molecular mechanism	Synergistic mechanism	Effect on SjD	References
H3K18la	A253 cells, ESS mice	unspecified	Promote	LC3B-II, Atg5, Beclin-1 ↑, p62 ↓	Inflammatory response ↑, lymphocytes infiltration↑	Drive SjD progression	(66)
U0126/Rapa	SjD patients, ESS mice, HSGECs	ERK1/2-mTOR ↓	Promote	LC3, Beclin-1 ↑, p62 ↓	unspecified	Drive SjD progression	(68)
Sphingosine-1-phosphate	NOD mice	unspecified	Promote	LC3 ↑	Inflammatory response ↓, lymphocytes infiltration ↓, salivary secretion ↑	Suppress SjD progression	(69)
Superoxide dismutase 3	NOD DE mice	unspecified	Inhibit	ATG5 ↓	Inflammatory response ↓, lymphocytes infiltration ↓, Tear secretion volume ↑	Suppress SjD progression	(71)
Chloroquine	NOD DE mice	unspecified	Inhibit	LC3B-II, ATG5 ↓	Inflammatory response ↓, lymphocytes infiltration ↓, Tear secretion volume ↑	Suppress SjD progression	(73)
Artesunate combined with Metformin	NOD mice	PI3K/AKT ↑	Inhibit	LC3, LC3-II/LC3-I ↓, p62 ↑	Salivary secretion ↑	Suppress SjD progression	(76)
Blueberry leaf extract	NOD mice	unspecified	Inhibit	LC3 ↓	Inflammatory response ↓	Suppress SjD progression	(77)

↑, Increase/Activate/Enhance; ↓, Decrease/Lower/Inhibit.

(“Unspecified” indicates that the original literature only reported the regulation of PCD effect molecules or inflammatory factors by the intervention measures, but did not further clarify the upstream signaling pathways. Such studies mainly focus on phenotypic validation and have not yet conducted mechanism analysis at the pathway level. This suggests that subsequent research needs to supplement pathway validation experiments. It should be noted that “Unspecified” does not mean that the intervention measure does not have signal pathway regulation, but reflects the current insufficient depth of mechanism analysis in the research).

## 4 Pyroptosis and SjD

### 4.1 Pyroptosis and its molecular mechanism

Pyroptosis is an inflammatory form of PCD mediated by gasdermin family proteins (78). The hallmark of this process is inflammatory cell necrosis, characterized by cell swelling, membrane perforation, and rupture. This leads to the massive release of pro-inflammatory cytokines, including IL-1β and IL-18, thereby triggering a robust inflammatory response (79). The mechanism of pyroptosis primarily involves two pathways: the canonical pathway, which depends on caspase-1, and the non-canonical pathway, which depends on caspase-4, caspase-5, and caspase-11. In the canonical pathway, upon infection by pathogens (e.g., bacteria or viruses) or stimulation by endogenous danger signals, intracellular pattern recognition receptors (PRRs) are activated. These receptors then recruit the adaptor protein ASC (apoptosis-associated speck-like protein containing a CARD) and pro-caspase-1 to form a multi-protein complex known as the inflammasome, which promotes caspase-1 activation. Once activated, caspase-1 cleaves gasdermin D (GSDMD), generating the active N-terminal fragment of GSDMD (GSDMD-NT) (80). In the non-canonical pathway, lipopolysaccharide (LPS) stimulation directly binds to and activates caspase-4, caspase-5, and caspase-11. These activated caspases induce pyroptosis and membrane lysis by cleaving GSDMD. Moreover, they can activate caspase-1 via the NLRP3 inflammasome, ultimately promoting the production and release of IL-1β and IL-18 (81).

### 4.2 Relationship between pyroptosis and SjD

Abnormal activation of the NLRP3 inflammasome may be closely related to the pathogenesis of SjD (82). In the LG and SG of SjD patients, the expression of NLRP3 inflammasome components is enhanced, suggesting that its abnormal activation may drive local innate immune inflammation and glandular dysfunction by mediating pyroptosis and releasing inflammatory factors such as IL-1β and IL-18 (83). During the progression of SjD, decreased expression of AQP5 induces massive production of ROS, which in turn activates the NLRP3/GSDMD complex, triggers caspase-1 cleavage, and ultimately induces cell pyroptosis (84). Type I interferons (IFN-α/β) may mediate NLRP3 inflammasome-related pyroptosis in SGECs, thereby participating in the destruction of target tissues (85). In patients with severe SjD, impaired DNA degradation leads to abnormal cytoplasmic DNA accumulation, which subsequently activates the NLRP3 inflammasome. This activation is reflected by elevated serum levels of cell-free DNA (cf-DNA), IL-18, and ASC. Collectively, these findings suggest the occurrence of inflammasome-mediated, caspase-1-dependent pyroptosis in patients with SjD (86). In patients with SjD, the expression levels of IL-1β, caspase-1, and ASC are significantly elevated, all of which are involved in NLRP3 inflammasome activation. Moreover, the expression levels of IL-1β and IL-18 are significantly correlated with the SjD Damage Index (SSDDI), suggesting that NLRP3 inflammasome-mediated inflammatory responses may contribute to the pathogenesis of SjD (87). The significantly elevated expression of the NLRP3 inflammasome and its downstream factors caspase-1, IL-1β, and IL-18 in patients with SjD-DE points to a role for the NLRP3 inflammasome in driving the initiation and progression of inflammatory processes in this condition (88).

### 4.3 Therapeutic potential of targeting pyroptosis

Excessive activation of pyroptosis is closely related to the damage of SG epithelial cells, lymphocyte infiltration and the amplification of autoimmune responses. It has become an emerging hotspot in the study of the pathogenesis of SjD. Therefore, inhibiting the abnormal pyroptosis in SjD is expected to become a highly promising therapeutic strategy. Studies have found that Iguratimod, P2X7 receptor antagonists, and some active components of TCM such as salvianolic acid B, ruscogenin, forsythiaside A, oridonin, rutin, total glucosides of paeony, and paeoniflorin, can inhibit pyroptosis, reduce inflammatory responses and lymphocyte infiltration, and restore glandular secretion function, thereby improving the dry mouth and dry eye symptoms of SjD. (The mechanism of the effect of regulating pyroptosis on SjD is shown in **Figure 4**).

**Figure 4:**
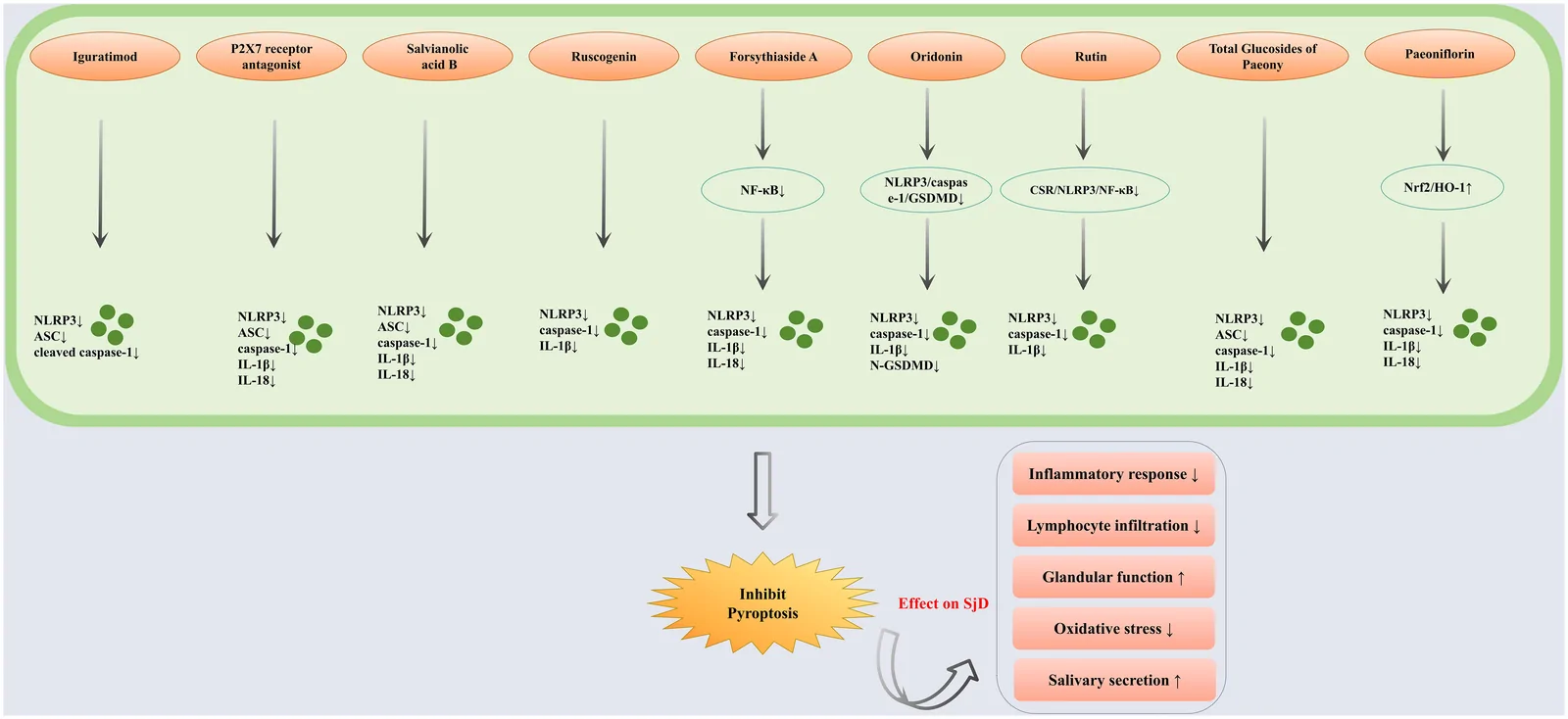
The mechanism of the effect of regulating pyroptosis on SjD.

Iguratimod (IGU) has anti-inflammatory and immunomodulatory effects and is commonly used to treat autoimmune diseases such as SjD (89). IGU downregulates the expression of NLRP3, ASC, and cleaved caspase-1, thereby blocking NLRP3 inflammasome activation and inhibiting pyroptosis in human submandibular gland (HSG) cells. Additionally, it reduces the expression of IL-1β, IL-6, and IL-18, thereby alleviating the inflammatory response. Furthermore, IGU inhibits lymphocyte infiltration in the glandular tissues of NOD mice and increases salivary secretion (90). However, the conclusions at the cellular level are based on immortalized HSG cell lines, which have limited physiological relevance; the results from NOD mice are closer to the *in vivo* environment, but species differences still limit their direct application to clinical practice. The P2X7 receptor (P2X7R) is an ATP-gated, non-selective cation channel that promotes inflammatory responses. P2X7 receptor antagonists down-regulate the expression of NLRP3, ASC, caspase-1, IL-1β, and IL-18, thereby inhibiting NLRP3 inflammasome activation and subsequently suppressing pyroptosis of submandibular gland (SMG) epithelial cells. Additionally, these antagonists alleviate inflammatory responses and lymphocyte infiltration, ultimately improving glandular function and increasing salivary secretion in NOD mice (91). Among them, the cell-level conclusions are based on SMG epithelial cells, and the physiological relevance is limited by the cell model; the results of NOD mice are limited by species differences. Ubiquitin-specific protease 5 (USP5) has been identified as a novel regulator of the NLRP3 inflammasome in the pathogenesis of SjD. In the SMG of NOD mice, USP5 expression is significantly downregulated. Salvianolic acid B (SalB) increases USP5 levels, which in turn reduces the expression of NLRP3, ASC, caspase-1, IL-1β, and IL-18, thereby inhibiting NLRP3 inflammasome activation and subsequently suppressing pyroptosis in primary submandibular gland cells (pSGC). Furthermore, SalB decreases the expression of inflammatory factors including TNF-α and IL-6, alleviates local inflammatory responses and lymphocyte infiltration, and ultimately improves the SG index and enhances salivary secretion in NOD mice (92). However, pSGC is a primary cell, which is closer to the physiological state than immortalized cell lines, but *in vitro* culture still cannot fully reflect the *in vivo* microenvironment; the results of NOD mice are limited by species differences. Ruscogenin (RUS) is a natural steroid saponin component, and it is widely used in the treatment of chronic inflammatory diseases (93). RUS downregulates the expression of NLRP3, caspase-1, and IL-1β, thereby inhibiting NLRP3 inflammasome activation and subsequently suppressing pyroptosis of SMG acinar cells. Additionally, it reduces the expression of pro-inflammatory cytokines such as TNF-α, IL-6, and IL-17, thereby alleviating the inflammatory response. Furthermore, RUS increases AQP5 expression, improves glandular function, and ultimately alleviates SMG pathological damage and enhances salivary secretion in NOD mice (94). The conclusions drawn at the cellular level are limited by the SMG acinar cell model, and the results for NOD mice are limited by species differences. Forsythiaside A (FA) is an active glycoside component extracted from the Chinese herb *Forsythia suspensa* (Thunb.) Vahl, and it possesses immunomodulatory and anti-inflammatory properties (95). FA promotes the degradation of GRP78, downregulates p65 expression, and inhibits the NF-κB signaling pathway. This leads to reduced expression of NLRP3, caspase-1, IL-1β, and IL-18, thereby suppressing NLRP3 inflammasome activation. Consequently, FA inhibits pyroptosis in primary submandibular gland cells (pSGC), alleviates the inflammatory response in NOD mice, and increases salivary secretion (96). However, pSGC is a type of primary cell, and further validation is required in an *in vivo* model; the results obtained from NOD mice are limited by species differences. Oridonin (Ori) is a terpenoid active component derived from the Chinese herb *Rabdosia rubescens* (Hemsl.) Hara. It exhibits good anti-inflammatory activity (97). A study has found that Ori down-regulates the expression of NLRP3, caspase-1, IL-1β, and N-GSDMD, thereby inhibiting the NLRP3/caspase-1/GSDMD signaling pathway and suppressing pyroptosis in human corneal epithelial cells (HCE-T). Furthermore, Ori reduces the corneal fluorescein staining score and increases tear secretion in NOD mice (98). However, HCE-T is an immortalized cell line with limited physiological relevance; the results obtained in NOD mice are also limited by species differences. Rutin (RUT) is a natural flavonoid compound that possesses various biological activities such as antioxidant, anti-inflammatory and immunomodulatory properties (99). RUT downregulates the expression of CSR, NLRP3, and p-NF-κB p65, thereby inhibiting the CSR/NLRP3/NF-κB signaling pathway. This leads to reduced expression of caspase-1 and IL-1β, which in turn suppresses pyroptosis in CaR-overexpressing primary submandibular gland cells (over-CaR-PSGs). Additionally, RUT increases the levels of superoxide dismutase (SOD) and glutathione peroxidase (GSH-Px), while reducing malondialdehyde (MDA) content, thereby inhibiting oxidative stress. It also decreases the expression of pro-inflammatory cytokines such as TNF-α, IL-1β, and IL-6, alleviating the inflammatory response. Ultimately, RUT reduces the SG index, improves glandular function, and increases salivary secretion in NOD mice (100). Since over-CaR-PSGs are primary cells, they are closer to the physiological state but still require *in vivo* verification; the results of NOD mice are limited by species differences. Total Glucosides of Paeony (TGP) possesses anti-inflammatory and immunomodulatory properties, making them applicable for the treatment of various autoimmune diseases (101). TGP reduces the expression of NLRP3, ASC, caspase-1, IL-1β, and IL-18, thereby inhibiting NLRP3 inflammasome activation and suppressing pyroptosis of SG cells. Additionally, it reduces lymphocyte infiltration, increases AQP5 expression, enhances glandular function, and ultimately increases salivary secretion in NOD mice (102). However, the conclusions drawn at the cellular level in this study are limited by the SG cell model, and the results for NOD mice are limited by species differences. Paeoniflorin (PF) possesses various biological activities such as anti-inflammatory, immunomodulatory and antioxidant properties (103). PF upregulates Nrf2 and HO-1 expression, thereby activating the Nrf2/HO-1 pathway. This leads to reduced expression of NLRP3, caspase-1, IL-1β, and IL-18, which in turn inhibits NLRP3 inflammasome activation and suppresses pyroptosis of SG cells. Concurrently, PF increases the proportion of Tregs while decreasing the proportion of Th1 cells, thereby improving the Th1/Treg immune balance. Furthermore, PF enhances the expression of ZO-1 and AQP5, improving glandular function in NOD mice. Additionally, it reduces lymphocyte infiltration in the glands and increases salivary secretion (104). However, in SjD patients and NOD mouse models, the NLRP3 inflammasome was significantly activated, but the sample size and heterogeneity of the patient samples still need to be verified; the conclusions at the cellular level are limited by the SG cell model; the results of the NOD mice are limited by species differences. (The mechanism of the effect of regulating pyroptosis on SjD is summarized in **Table 3**).

**Table 3:** Summary of the mechanisms of the effect of regulating pyroptosis on SjD.

Intervention	Model	Signaling pathway	Pyroptosis regulation	Molecular mechanism	Synergistic mechanism	Effect on SjD	References
Iguratimod	HSG cells, NOD mice	unspecified	Inhibit	NLRP3, ASC, cleaved caspase-1 ↓	Inflammatory response ↓, lymphocyte infiltration ↓, salivary secretion ↑	Suppress SjD progression	(90)
P2X7 receptor antagonist	SMG epithelial cells, NOD mice	unspecified	Inhibit	NLRP3, ASC, caspase-1, IL-1β, IL-18 ↓	Inflammatory response ↓, lymphocyte infiltration ↓, glandular function ↑	Suppress SjD progression	(91)
Salvianolic acid B	pSGC cells, NOD mice	unspecified	Inhibit	NLRP3, ASC, caspase-1, IL-1β, IL-18 ↓	Inflammatory response ↓, lymphocyte infiltration ↓, glandular function ↑, salivary secretion ↑	Suppress SjD progression	(92)
Ruscogenin	SMG acinar cells, NOD mice	unspecified	Inhibit	NLRP3, caspase-1, IL-1β ↓	Inflammatory response ↓, gland function ↑, salivary secretion ↑	Suppress SjD progression	(94)
Forsythiaside A	pSGC cells, NOD mice	NF-κB↓	Inhibit	NLRP3, caspase-1, IL-1β, IL-18 ↓	Inflammatory response ↓, salivary secretion ↑	Suppress SjD progression	(96)
Oridonin	HCE-T, NOD mice	NLRP3/caspase-1/GSDMD↓	Inhibit	NLRP3, caspase-1, IL-1β, N-GSDMD ↓	Corneal fluorescein staining score ↓, tear secretion ↑	Suppress SjD progression	(98)
Rutin	over-CaR-PSGs, NOD mice	CSR/NLRP3/NF-κB↓	Inhibit	NLRP3, caspase-1, IL-1β ↓	Oxidative stress ↓, inflammatory response ↓, salivary gland index ↓, salivary secretion ↑	Suppress SjD progression	(100)
Total Glucosides of Paeony	SG cells, NOD mice	unspecified	Inhibit	NLRP3, ASC, caspase-1, IL-1β, IL-18 ↓	Lymphocyte infiltration ↓, gland function ↑, salivary secretion ↑	Suppress SjD progression	(102)
Paeoniflorin	SG cells, NOD mice	Nrf2/HO-1↑	Inhibit	NLRP3, caspase-1, IL-1β, IL-18 ↓	Restore the Th1/Treg balance, Gland function ↑, lymphocyte infiltration ↓, salivary secretion ↑	Suppress SjD progression	(104)

↑, Increase/Activate/Enhance; ↓, Decrease/Lower/Inhibit.

(“Unspecified” indicates that the original literature only reported the regulation of PCD effect molecules or inflammatory factors by the intervention measures, but did not further clarify the upstream signaling pathways. Such studies mainly focus on phenotypic validation and have not yet conducted mechanism analysis at the pathway level. This suggests that subsequent research needs to supplement pathway validation experiments. It should be noted that “Unspecified” does not mean that the intervention measure does not have signal pathway regulation, but reflects the current insufficient depth of mechanism analysis in the research).

## 5 Ferroptosis and SjD

### 5.1 Ferroptosis and its molecular mechanism

Ferroptosis is a form of iron-dependent, oxidative stress-driven regulated cell death characterized by dysregulated metabolism of iron, polyunsaturated fatty acids (PUFAs), and lipids (105). Imbalanced intracellular iron metabolism serves as the key trigger for this process. Specifically, transferrin binds to its receptor to facilitate the uptake of extracellular iron. The internalized ferrous ions (Fe^2+^) then participate in various cellular physiological activities through a series of metabolic processes (106). Excess Fe^2+^ catalyzes the production of highly toxic hydroxyl radicals via the Fenton reaction, thereby inducing lipid peroxidation (LPO), which is the core step of the ferroptosis execution. Under the action of arachidonic acid epoxygenase and other enzymes, saturated fatty acids, monounsaturated fatty acids, and polyunsaturated fatty acids within cells are easily attacked by free radicals, undergo oxidative modification, and generate LPO products such as malondialdehyde (MDA) and 4-hydroxynonenal (4-HNE). The continuous accumulation of these products damages the structural and functional integrity of the cell membrane, causing cell swelling and rupture, and ultimately leading to ferroptosis (107).

### 5.2 Relationship between ferroptosis and SjD

Ferroptosis of SGECs plays a crucial role in glandular dysfunction caused by SjD. CD4^+^ T cells are the main source of IFN-γ secretion in the SG, and this cytokine induces ferroptosis of SGECs and inhibits AQP5 expression, thereby damaging SG function (108). IFN-γ activates STAT1 phosphorylation and downregulates solute carrier family 3 member 2 (SLC3A2), glutathione (GSH), and GPX4 expression, thereby promoting ferroptosis in SGECs and further aggravating the symptoms of SjD (109). In SGECs of SjD patients, GPX4 expression is downregulated, leading to the accumulation of lipid reactive oxygen species (ROS). This, in turn, activates STAT4 phosphorylation and nuclear translocation. Phosphorylated STAT4 binds to the promoter of AQP5 and inhibits its transcription, ultimately causing glandular dysfunction and decreased salivary secretion (110). Furthermore, ferroptosis-related proteins and gene markers are of great significance in the diagnosis and pathological assessment of SjD. Studies have shown that the expression of ferroptosis-related proteins (such as CP, TF, C5, and C9) in the plasma exosomes of SjD patients is downregulated, suggesting epithelial cell lesions, and this change is more sensitive in plasma exosomes than in plasma (111). Meanwhile, an integrated analysis identified ferroptosis-related genes such as TBK1, SLC1A4, PIK3CA, ENO3, EGR1, and ATG5, which could serve as potential diagnostic markers for SjD and revealed their association with the immune microenvironment (112).

### 5.3 Therapeutic prospects of targeting ferroptosis

Ferroptosis of SGECs plays a crucial role in the pathological process of SjD, directly leading to glandular dysfunction and decreased secretion function. Therefore, inhibiting ferroptosis represents a new and important therapeutic direction for SjD. Studies have found that ADSC-HExos, melatonin, autologous serum, as well as active ingredients of TCM such as genistein, apigenin, and artesunate, can exert anti-SjD effects by inhibiting ferroptosis. (The mechanism of the effect of regulating ferroptosis on SjD is shown in **Figure 5**).

**Figure 5:**
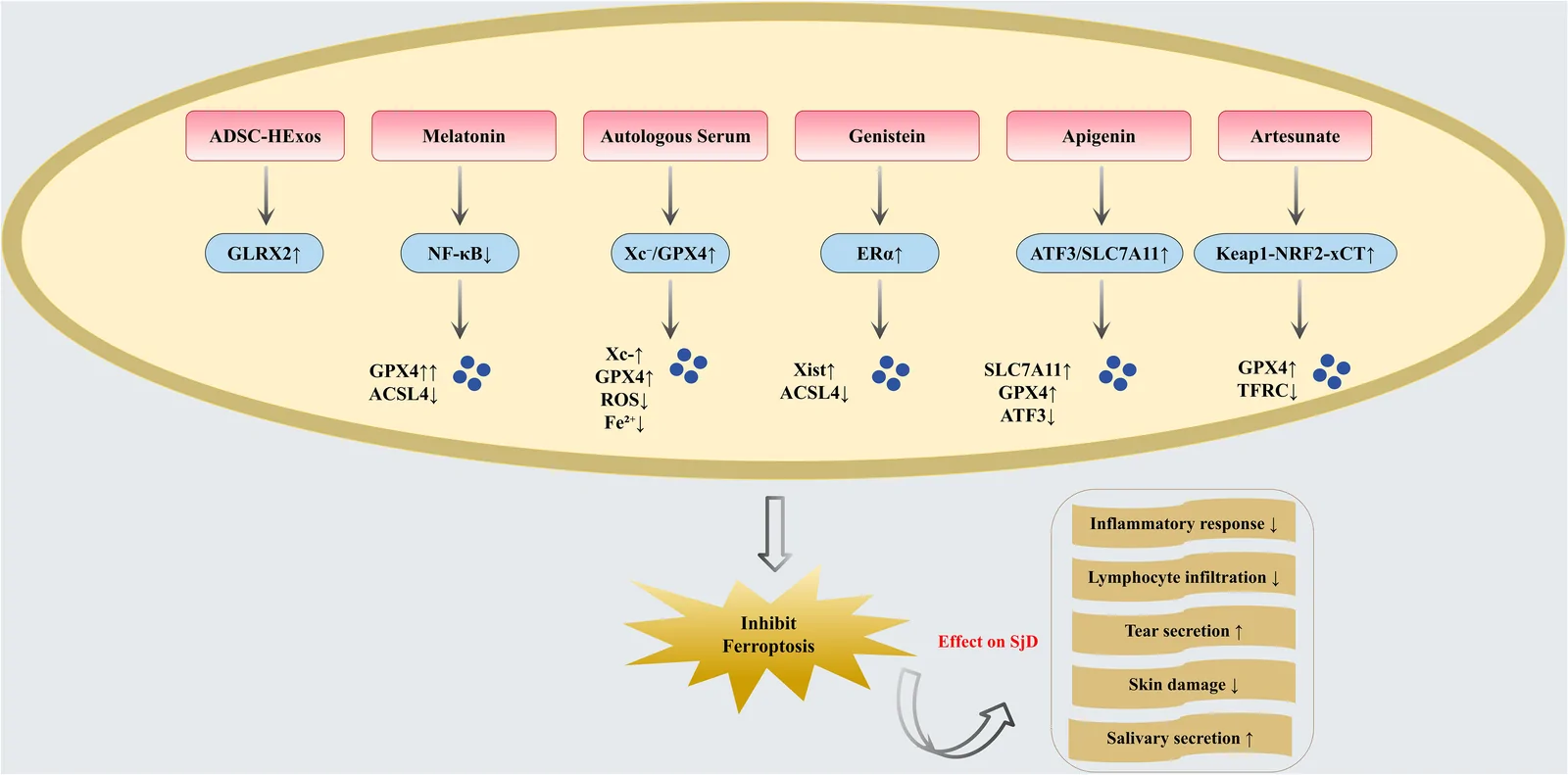
The mechanism of the effect of regulating ferroptosis on SjD.

Approximately 15% of patients with SjD will experience skin damage symptoms (113). Exosomes derived from adipose-derived stem cells (ADSC-HExos) have the potential to repair diabetic wounds (114). Glutaredoxin 2 (GLRX2) is abundantly present in various tissues and exhibits multiple pleiotropic effects, including antioxidant and anti-inflammatory functions (115). ADSC-HExos upregulate GLRX2 expression, inhibit ferroptosis in human epidermal keratinocytes (HaCaT cells), reduce reactive oxygen species (ROS) deposition, and increase type I collagen expression. They also alleviate skin damage in NOD mice and decrease the expression of TNF-α, IL-1β, and IL-6, thereby attenuating the inflammatory response (116). However, HaCaT is an immortalized cell line with limited physiological relevance; the results obtained in NOD mice are also limited by species differences. Melatonin (MLT) upregulates NRF2 and HO-1 expression, thereby activating the NRF2/HO-1 pathway, while concurrently downregulating p-p65 expression to inhibit the NF-κB pathway. It also increases GPX4 expression and reduces ACSL4 expression, thereby inhibiting ferroptosis in HSGE. Additionally, MLT reduces the expression of pro-inflammatory cytokines including TNF-α, IL-1β, IL-6, and IL-17, alleviating the inflammatory response. In NOD mice, MLT reduces the spleen index, alleviates glandular lymphocyte infiltration, improves SG function, and increases salivary secretion (117). However, HSGE represents immortalized cell lines, which have limited physiological relevance; the results obtained in NOD mice are limited by species differences; the oral bioavailability of MLT is low, and the exposure levels in target organs still need to be studied. Autologous serum (AS) has a good therapeutic effect in the treatment of SjD-DE (118). AS from SjD patients activates the Xc^-^/GPX4 signaling pathway, upregulates Xc^-^ and GPX4 expression, reduces ROS and Fe^2+^ levels, inhibits ferroptosis in corneal epithelial cells (HCE-T), alleviates lipid peroxidation, improves mitochondrial damage, enhances cell survival and migration, and increases tear secretion (119). However, HCE-T is an immortalized cell line with limited physiological relevance; AS, on the other hand, comes from the patient’s own serum, and issues related to batch consistency and standardization still need to be addressed. Genistein (GEN) is a natural isoflavone compound that demonstrates the potential to alleviate symptoms of various autoimmune diseases (120). GEN activates the estrogen receptor-α (ERα) signaling pathway, upregulates X-inactive specific transcript (Xist) expression, and downregulates ACSL4 expression, thereby inhibiting ferroptosis in SGECs, reducing lymphocyte infiltration, improving glandular function in NOD mice, and increasing salivary secretion (121). However, SGECs are immortalized cell lines with limited physiological relevance; the results obtained in NOD mice are also limited by species differences. Apigenin (AP) can upregulate the expression of estrogen receptor alpha (ERα), increase the expression of AQP5 in SGECs, restore the glandular function of NOD mice, and increase salivary secretion (122). AP upregulates ERα expression, thereby activating the ATF3/SLC7A11 signaling pathway, upregulating solute carrier family 7 member 11 (SLC7A11) and GPX4 expression, downregulating activating transcription factor 3 (ATF3) expression, inhibiting ferroptosis in SGECs, reducing glandular lymphocyte infiltration, increasing AQP5 expression, improving glandular function in NOD mice, and increasing salivary secretion (123). However, SGECs are immortalized cell lines with limited physiological relevance; the results obtained in NOD mice are also limited by species differences. Artesunate (ART) can inhibit the Th17 response by suppressing glycolysis mediated by interferon regulatory factor 4 (IRF4), and increase salivary secretion in NOD mice (124). Furthermore, ART enhances the expression of nuclear factor erythroid 2-related factor 2 (NRF2) and SLC7A11, reduces the expression of Kelch-like ECH-related protein 1 (Keap1), activates the Keap1-NRF2-xCT signaling pathway, upregulates GPX4 expression, downregulates transferrin receptor (TFRC) expression, and inhibits ferroptosis in SGECs. Additionally, it alleviates pathological damage to glandular tissues in NOD mice, increases AQP5 expression, and enhances salivary secretion (125). However, SGECs are immortalized cell lines with limited physiological relevance; the results obtained from NOD mice are limited by species differences; the targeted organ delivery and safe dosage of ART still require further research. (The mechanism of the effect of regulating ferroptosis on SjD is summarized in **Table 4**).

**Table 4:** Summary of the mechanisms of the effect of regulating ferroptosis on SjD.

Intervention	Model	Signaling pathway	Ferroptosisregulation	Molecular mechanism	Synergistic mechanism	Effect on SjD	References
ADSC-HExos	HaCaT cells, NOD mice	unspecified	Inhibit	unspecified	Skin damage ↓, inflammatory response ↓	Suppress SjD progression	(116)
Melatonin	HSGE, NOD mice	NF-κB ↓	Inhibit	GPX4 ↑, ACSL4 ↓	Inflammatory response ↓, lymphocyte infiltration ↓, salivary secretion ↑	Suppress SjD progression	(117)
Autologous Serum	HCE-T cells, SjD patients	Xc^−^/GPX4 ↑	Inhibit	Xc^-^, GPX4 ↑, ROS, Fe^2+^ ↓	Tear secretion ↑	Suppress SjD progression	(119)
Genistein	SGECs, NOD mice	ERα↑	Inhibit	Xist ↑, ACSL4 ↓	Lymphocyte infiltration ↓, salivary secretion ↑	Suppress SjD progression	(121)
Apigenin	SGECs, NOD mice	ATF3/SLC7A11 ↑	Inhibit	SLC7A11, GPX4 ↑, ATF3 ↓	Lymphocyte infiltration ↓, salivary secretion ↑	Suppress SjD progression	(123)
Artesunate	SGECs, NOD mice	Keap1-NRF2-xCT ↑	Inhibit	GPX4 ↑, TFRC ↓	Oxidative stress ↑, pathological damage of SG ↑, salivary secretion ↑	Suppress SjD progression	(125)

↑, Increase/Activate/Enhance; ↓, Decrease/Lower/Inhibit.

(“Unspecified” indicates that the original literature only reported the regulation of PCD effect molecules or inflammatory factors by the intervention measures, but did not further clarify the upstream signaling pathways. Such studies mainly focus on phenotypic validation and have not yet conducted mechanism analysis at the pathway level. This suggests that subsequent research needs to supplement pathway validation experiments. It should be noted that “Unspecified” does not mean that the intervention measure does not have signal pathway regulation, but reflects the current insufficient depth of mechanism analysis in the research).

## 6 Discussion

The pathogenesis of SjD is complex, and various forms of PCD, such as apoptosis, autophagy, pyroptosis, and ferroptosis, play significant roles in the onset and progression of this disease. By regulating these forms of PCD, important effects can be exerted on the onset, treatment, and prognosis of SjD. This article systematically summarizes and analyzes the existing relevant literature, and the main findings are as follows: (1) In terms of regulating the forms of PCD, current research focuses on apoptosis, autophagy, pyroptosis, and ferroptosis. Among these, inhibiting apoptosis, pyroptosis, and ferroptosis is considered an important strategy for treating SjD. Notably, two independent studies have respectively revealed the potential value of a miR-155-5p inhibitor in treating SjD from the perspective of bidirectional regulation of apoptosis. Autophagy has a bidirectional regulatory property, and its role in SjD varies depending on whether it is activated or inhibited (protection or damage). (2) Regarding the intervention subjects for regulating PCD, the main entities include miRNAs, protein molecules (such as B7-H3, CTRP3, and β-arrestin2), exosomes (such as SHED-exos and ADSC-HExos), stem cell (such as ME-MSCs), chemical drugs (such as chloroquine, metformin, and iguratimod), and various active components and compound formulas of TCM (such as AS-OD, Salvianolic acid B, Ruscogenin, Forsythiaside A, and Oridonin). These interventions exert multi-mechanism and multi-target therapeutic effects on SjD by regulating apoptosis, autophagy, pyroptosis, or ferroptosis. Among these, the regulation of PCD by active components of TCM for the treatment of SjD shows great potential. (3) In terms of signaling pathways, various intervention methods can activate or inhibit pathways such as NF-κB, PI3K/AKT, and JAK/STAT, forming a complex signaling network. Among these, the regulation of the NF-κB pathway is relatively well-characterized. For example, upregulating miR-155-5p can activate this pathway and promote apoptosis, whereas downregulating miR-155-5p, miR-34a, or miR-223-3p, or upregulating miR-23b-3p, can inhibit this pathway and produce an anti-apoptotic effect. Additionally, forsythiaside A can inhibit the NF-κB pathway and alleviate pyroptosis. Two other studies found that Melatonin inhibits the IL-6/STAT3 pathway, and quercetin inhibits the JAK2/STAT3 pathway; both can inhibit apoptosis. Artemisinin combined with Metformin can activate the PI3K/AKT pathway and inhibit autophagy, while U0126 or Rapa can inhibit the ERK1/2-mTOR pathway and promote autophagy. Overall, these pathways interweave with each other, forming a signaling network that regulates inflammation, immunity, and various forms of PCD in SjD, thereby providing potential targets for targeted treatment of SjD. (4) In terms of research types, the cell models used mainly include SGECs, HSGE, and HSG. Some studies also use other cell lines. For example, AS-OD acts on A253 cells; oridonin acts on HCE-T cells; ruscogenin acts on SMG acinar cells; iguratimod acts on SMG epithelial cells; salvianolic acid B and forsythiaside A act on pSGC cells; rutin acts on over-CaR-PSGs cells; ADSC-HExos acts on HaCaT cells; and autologous serum acts on HCE-T cells. In terms of animal models, NOD mice and NOD-DE mice are mainly used, while epigallocatechin gallate acts on MRL/lpr mice to study SjD-related autoimmune SG inflammation. It is worth noting that downregulating miR-223-3p expression, upregulating miR-1207-5p/miR-4695-3p expression, and applying interventions such as B7-H3, CTRP3, IL-11, and U0126/Rapa have demonstrated good therapeutic potential in SjD patients. (5) Moreover, the same intervention or drug often has multiple regulatory effects on PCD. For example, iguratimod can inhibit apoptosis and pyroptosis; melatonin inhibits apoptosis and ferroptosis; artemisinin alone inhibits ferroptosis, while the combination of artemisinin and metformin inhibits autophagy. SHED-exos can simultaneously regulate multiple PCD pathways, including apoptosis, ferroptosis, oxidative death, and necroptosis. This multi-PCD regulatory effect may be related to the high convergence and crosstalk of upstream signaling pathways, the direct interaction and conversion between different PCD pathways, and the multi-target pharmacological properties of the drug itself. (6) Regarding the specific therapeutic effects on SjD, current studies have different focuses. Most intervention strategies mainly aim to improve dry mouth symptoms. In addition, ADSC-HExos improves skin damage by inhibiting ferroptosis; downregulating miR-223-3p expression can inhibit apoptosis; blueberry leaf extract, chloroquine, and SOD3 inhibit autophagy; autologous serum inhibits ferroptosis; and oridonin inhibits pyroptosis. These interventions can all improve dry eye symptoms. The specific therapeutic effects on SjD vary, which may be related to the intrinsic fragility of cell types, the specificity of the organ microenvironment, and the distribution of pharmacological targets of the intervention measures. (7) Based on the summary of the above PCD execution mechanism, it is necessary to further examine its upstream triggering factors and regulatory hubs in order to construct a more complete pathogenic model. Recent studies have shown that CCR1^hi/CCL5^hi macrophages recruit CCL5^hi T cells and B cells to infiltrate salivary glands through CCL5-mediated chemotaxis, forming an important upstream driving factor of the glandular inflammation microenvironment in SjD. This immune infiltration process occurs prior to or concurrently with the activation of PCD, suggesting that PCD is not merely an intrinsic event of glandular epithelium, but is triggered and amplified under the drive of the immune microenvironment (126). At the same time, LAMP3, as a regulatory hub connecting IFN/TLR7 signals and lysosomal dysfunction, is induced to be expressed in salivary gland epithelial cells by type I interferons and further amplified by TLR7 activation; abnormal upregulation of LAMP3 promotes the lysosomal degradation of AQP5 and NKCC1, leading to impaired glandular secretion function, and simultaneously drives the release of DAMPs through lysosomal exocytosis and mediates the release of self-antigens via extracellular vesicles, and participates in lysosome-dependent cell death (127). Therefore, the CCR1/CCL5 axis and LAMP3 provide upstream explanations from the two levels of immune triggering and lysosomal regulation for the PCD execution mechanism. Incorporating these two pathways into the pathogenic model helps to construct a complete chain of “immune triggering - lysosomal regulation - PCD execution - glandular damage”, rather than focusing solely on the terminal events of the downstream death pathway.

Furthermore, to address the shortcomings of the original manuscript, which focused excessively on the listing of intervention measures and lacked critical comprehensive analysis, this article further integrates the existing intervention measures from three aspects: evidence level, depth of functional validation, and drug development potential. The construction basis of this evaluation framework is as follows: The degree of independent verification is determined by the number of verification teams or models as the grading criterion; the depth of functional experiments is judged by whether there are causal verification methods such as knockout, overexpression, and restoration experiments; the potential for drug development is evaluated by whether there are in vivo data, clinical-stage inhibitors, pharmacokinetic data, and standardized production processes. (1) In terms of independent validation, the targets that have been truly verified by multiple independent research groups still account for a minority in the existing studies, such as miR-155-5p, NLRP3 inflammasome, NF-κB pathway, and JAK/STAT pathway. While the active ingredients of many traditional Chinese medicines, some miRNAs, and exosomes mostly come from a single research group or a single model, and lack independent repetition. (2) In terms of functional experimental support for the proposed mechanisms, some studies have surpassed the simple correlation of protein expression through knockout, overexpression, inhibitors or restoration experiments, such as the verification of NLRP3 inflammasome by inhibitors or knockout, and the functional experiments of miR-155-5p in both positive and negative directions. However, for B7-H3, CTRP3, and most active ingredients of traditional Chinese medicines, the current experiments mainly focus on expression changes and overexpression, lacking knockout or restoration experiments, and the causal relationship still needs to be further confirmed. (3) In terms of drug development potential, based on the comprehensive evidence level, depth of functional validation, and in vivo data, this review believes that the NF-κB pathway, NLRP3 inflammasome, JAK/STAT pathway, and miR-155-5p are currently the most promising candidate targets for transformation. Among them, the NLRP3 inflammasome has multiple inhibitors entering the clinical research stage, with the shortest transformation path. The JAK/STAT pathway, due to the use of JAK inhibitors such as tofacitinib and baricitinib in other autoimmune diseases, has a clear basis for drug development, while miR-155-5p is a potential candidate for nucleic acid drug development. In contrast, although most active ingredients of traditional Chinese medicines have preliminary in vivo data, they lack independent repetition and pharmacokinetic data, and are limited by the lack of standardization in separation, characterization, potency determination, and batch release. The transformation cycle is relatively long. In summary, in the near future, priority should be given to promoting the preclinical transformation research of inhibitors related to NF-κB, NLRP3, and JAK/STAT pathways, while miR-155-5p inhibitors and engineered exosomes need to solve the problems of delivery, standardization, and safety issues before advancing to clinical trials in the medium to long term.

Although the existing research has made significant progress, there are still some shortcomings. For each of these shortcomings, this paper further proposes corresponding research directions and countermeasures, with the aim of providing references for subsequent research. (1) There is a disconnect between disease models and clinical reality. Although immortalized cell lines and NOD mice facilitate the exploration of mechanisms and intervention screening, they cannot replicate the chronic inflammatory microenvironment and human heterogeneity of SjD patients, lacking several key pathological features. Firstly, the human-specific antigen presentation system is absent. The immortalized cell lines contain only a single cell type and lack professional antigen-presenting cells and the human HLA polymorphism background, making it impossible to fully replicate the processing and presentation of self-antigens such as Ro/SSA and La/SSB; the MHC molecules of NOD mice differ significantly from those of humans in structure, peptide binding preference, and are difficult to simulate the immune recognition mechanism of human self-antigens. Secondly, they cannot truly reproduce the co-activation mechanism of T and B cells. The onset of SjD depends on the coordinated activation of T and B cells in the glands and the germinal center reaction, which is a key factor for the production of autoantibodies, and neither of these models can accurately replicate the activation sequence, intensity, and molecular regulatory characteristics of this process. Finally, key pathological structures are missing. As the core structure for glandular damage and autoantibody production in SjD, the ectopic germinal center forms only partially in NOD mice, and immortalized cell lines completely fail to reflect this. These deficiencies result in the conclusion of current model studies only reflecting the controlled experimental responses of individual cells, with limited clinical reference value. Therefore, when citing specific research conclusions in this paper, the source of the model and the strength of the evidence have been simultaneously marked. Only through successive verification by humanized mice, humanized organs, and clinical trials can the gap between the model and clinical reality be gradually narrowed. In response to the aforementioned shortcomings, future research should focus on constructing a disease model with greater clinical relevance and establishing a time dynamic map of PCD. Construct more clinically relevant disease models, establish humanized mouse models (by transplanting patient PBMCs or SG tissues), develop 3D organoids of SG, LG, and skin, and co-culture them with immune cells. At the same time, a longitudinal study was conducted in NOD mice to draw a time-dependent change map of PCD markers during the disease progression, providing a temporal dimension basis for the verification of pharmacodynamic markers. (2) Research on the PCD mechanism is fragmented, mostly limited to a single death pathway, and lacks synchronous dynamic monitoring of apoptosis, autophagy, pyroptosis, and ferroptosis. Consequently, it remains difficult to reveal the crosstalk, transformation, and competitive balance mechanisms among these pathways. At the same time, the bidirectional regulatory characteristics of autophagy in SjD, that is, the specific conditions under which it exerts protective or pro-inflammatory damage in different conditions, still need to be systematically clarified. Therefore, by conducting a cross-analysis of the four types of PCD from an integrated perspective, we can select the NF-κB pathway or oxidative stress as the common node, elevating apoptosis, autophagy, pyroptosis, and ferroptosis from a parallel classification relationship to a hierarchical and biased regulatory network. On one hand, when the NF-κB pathway serves as the hub, it promotes pyroptosis by driving the NLRP3 inflammasome and pro-IL-1β transcription, simultaneously upregulating anti-apoptotic proteins such as Bcl-2 to inhibit apoptosis, and exerting context-dependent regulation on the sensitivity of autophagy and ferroptosis, thereby determining in the pathway selection level which death mode the cell will preferentially adopt under inflammatory stimulation. On the other hand, when oxidative stress is used as the common trigger factor, ROS is both the inducing signal for mitochondrial outer membrane permeabilization in apoptosis and the executive force for lipid peroxidation in ferroptosis, and can also activate the NLRP3 inflammasome through oxidative mtDNA to promote pyroptosis. Autophagy not only clears the source of ROS but also promotes ferroptosis through ferritin autophagy. Its protective and pro-death dual nature can only be consistently explained within the framework of the threshold and bias. Moderate ROS can be buffered by autophagy and antioxidant systems, and continuous accumulation will exceed the threshold and enter the irreversible death execution stage. Thus, by connecting cross-relation through a common hub and replacing simple parallelism with thresholds and biases, it not only helps to reveal the mutual regulation and competitive balance among the four types of PCD, enhances the theoretical depth of cross-analysis of PCD, but also provides a new framework for understanding the imbalance of PCD network and the relationship between inflammation persistence, tissue fibrosis in SjD. It also suggests that targeting NF-κB or oxidative stress may become a potential therapeutic direction. In response to the aforementioned shortcomings, future research should focus on simultaneously detecting multiple PCD indicators and constructing a regulatory node map. By synchronously detecting indicators of apoptosis, autophagy, pyroptosis, and ferroptosis, and using protein interaction networks and transcriptome sequencing to construct a regulatory node map, we can deeply explore the conditions and molecular mechanisms underlying the transformation from apoptosis to pyroptosis, thereby promoting the understanding of the PCD network and providing a mechanistic basis for multi-target intervention. This approach will also help to systematically clarify the conditions under which autophagy exerts protective versus pro-inflammatory effects in SjD. (3) Evidence for clinical translation is weak, and the translation pathway is not well established. Most intervention measures stop at the cellular or animal stage, and very few enter clinical trials. Current inclusion criteria rely on subjective symptoms such as dry mouth and dry eyes rather than PCD functional classification, which leads to efficacy being diluted by heterogeneity. Moreover, the contradiction between the administration route and target organ distribution is prominent: oral administration has low bioavailability, and there is a lack of standards for local formulations, resulting in severely insufficient drug concentrations in glandular tissues. In response to the aforementioned shortcomings, future research should focus on developing and validating non-invasive PCD markers to facilitate patient stratification and precise medication. By developing non-invasive PCD biomarkers in saliva and tears, patient stratification can be achieved, and clinical trials can be designed based on PCD classification (for example, using iguratimod in dry mouth cases dominated by apoptosis, and using orienedin in local treatment of dry eye syndrome dominated by balloon-like necrosis). These markers need to be verified in independent cohorts for their correlation with hard endpoints of glandular function, and standardized detection methods and reference intervals should be established. In addition, precisely adjust the administration regimens of old drugs such as chloroquine and melatonin, improve the oral bioavailability of natural compounds through nanocarriers, prodrug design or structural modification, conduct target organ pharmacokinetic studies, and develop new targeted delivery systems for the corneal epithelium or the ocular surface to directly address the three major obstacles of PCD marker deficiency, difficult target organ delivery, and low bioavailability. (4) The depth of exploration of multi-target drug mechanisms is insufficient. On the one hand, for complex intervention substances such as exosomes, research mostly remains at the descriptive level of “simultaneously regulating multiple PCD pathways,” lacking validation through specific knockdown of key miRNAs or proteins for functional dissection, making it difficult to identify the molecular switch that dominates the effect. On the other hand, although combination therapy (such as artemisinin combined with metformin) shows an apparent synergistic effect, the molecular linkage points and optimal ratio range for its interactive regulation of PCD pathways remain unexplored. In response to the aforementioned shortcomings, future research should focus on conducting in-depth analysis of the molecular mechanisms of multi-target intervention, optimizing the combination strategies, and promoting the standardization of exosome formulations. Utilize miRNA knockout or overexpression techniques to identify specific effector molecules in exosomes, adopt factor design or response surface methods to optimize drug combinations, and introduce single-cell sequencing technology to reveal the heterogeneity of PCD among different cell types. At the same time, it is necessary to simultaneously establish standardized processes for exosome isolation, characterization, potency determination, and batch release, clearly define their regulatory classification, in order to solve the transformation obstacle of the lack of standardization of exosome formulations. (5) The research on organ-specific PCD and symptom association is insufficient. Current research is overly concentrated on the mechanism of acinar cell death behind dry mouth, while the exploration of the PCD basis in other important target organs such as dry eyes, skin damage, arthritis, and even renal tubular acidosis is insufficient. More critically, there is still a lack of parallel, cross-organ comparative studies to determine whether the activation patterns of PCD in different organs of the same SjD patient show consistency or organ specificity. In response to the aforementioned shortcomings, future research should focus on expanding the research on organ-specific PCD and formulating local treatment strategies. Expand the research on organ-specific PCD and its symptoms, construct a distribution map of PCD activation in multiple organs, formulate local treatment strategies for dry eye and skin damage, and extend the research to severe complications such as neuropathy, renal tubular acidosis, and lymphoma transformation, providing a basis for targeted organ delivery and indication expansion.

Based on the above obstacles and countermeasures, there are differences in the feasibility of each transformation direction in terms of time. The division of transformation time levels in this article is as follows: The recent level refers to directions that have measurable clinical endpoints, can partially avoid systemic pharmacokinetic barriers through local administration, and do not require the establishment of new regulatory standards; the medium-term level refers to directions that require breakthroughs in formulation technology or the establishment of standardized processes, but have preliminary *in vivo* data support; the long-term level refers to directions with clear mechanism value, but still lack clinical-stage drugs or mature delivery systems, and require completing the concept verification first. Based on this, the transformation directions can be divided into the following three levels. (1) The recent level (1–3 years), the validation of saliva/tear PCD markers and the development of local delivery systems targeting the ocular surface/salivary glands have the greatest transformation potential. The basis for this determination is that these two types of directions directly act on measurable clinical endpoints (such as gland function indicators, tear secretion volume), and local administration can partially avoid systemic pharmacokinetic barriers, and can be advanced without waiting for new drug approval. (2) The medium-term level (3–5 years), the pharmacokinetic studies of natural compounds in target organs and the standardization of exosome formulations require long-term investment. The former relies on breakthroughs in formulation technology (such as nanocarriers, prodrug design), and the latter requires three prerequisite conditions: unified separation methods, the establishment of efficacy units, and clear regulatory classification. These two types of directions have preliminary *in vivo* data support, but the standardization and process bottlenecks have not been resolved. (3) The long-term level (5 years or more), multi-target optimization and organ-specific PCD maps have high mechanism value, but are still relatively far from clinical transformation. The basis for this determination is that these two types of directions still lack clinical-stage drugs or mature delivery systems, and need to complete concept verification and mechanism confirmation before entering the transformation path. In summary, through the above measures, researchers can systematically break through the key bottlenecks in current SjD research, including model disconnection, fragmentation of mechanisms, weak transformation capabilities, insufficient multi-target analysis, and lack of organ-specific research. This will lay a solid theoretical foundation and transformation basis for formulating personalized treatment strategies based on PCD classification, and ultimately achieve the clinical goals of improving symptoms in SjD patients, delaying disease progression, and reducing complications.

## Glossary

SG: Salivary glands
SMG: Submandibular gland
HSGECs: Human Salivary Gland Epithelial Cells
HSGE: Human Submandibular Gland Epithelial cells
HSG: Human Salivary Gland cells
SMG-C6: Submandibular Gland cell line C6
HCE-T: Human Corneal Epithelial cells
HaCaT: Human keratinocyte cell line
PCD: Programmed Cell Death
NF-κB: Nuclear Factor kappa-B
PI3K/AKT: Phosphatidylinositol 3-Kinase / Protein Kinase B
JAK/STAT: Janus Kinase / Signal Transducer and Activator of Transcription
NLRP3: NOD-like receptor family pyrin domain containing 3
GPX4: Glutathione Peroxidase 4
ACSL4: Acyl-CoA Synthetase Long-Chain Family Member 4
AQP5: Aquaporin 5
Bax: Bcl-2-associated X protein
Bcl-2: B-cell lymphoma 2
Caspase: Cysteine-aspartic acid protease
LC3: Microtubule-associated protein 1 light chain 3
mTOR: Mammalian Target of Rapamycin
MLT: Melatonin
IGU: Iguratimod
CQ: Chloroquine
ART: Artesunate
Met: Metformin
BLEx: Blueberry Leaf Extract
EGCG: Epigallocatechin Gallate
QUE: Quercetin
ZYD: Zengye Decoction
SalB: Salvianolic Acid B
RUS: Ruscogenin
FA: Forsythiaside A
Ori: Oridonin
RUT: Rutin
TGP: Total Glucosides of Paeony
PF: Paeoniflorin
AS: Autologous Serum
AS-OD: Astragalus-Salvia / Ophiopogon-Dendrobium combination
DP: Dendrobium Polysaccharide
GEN: Genistein
AP: Apigenin
NOD: Non-Obese Diabetic mice
NOD-DE: Non-Obese Diabetic mice with Dry Eye
MRL/lpr: MRL/MpJ-Faslpr mice
SjD: Sjögren's Disease
SjD-DE: Sjögren's Disease with Dry Eye
PBMC: Peripheral Blood Mononuclear Cell
MSC: Mesenchymal Stem Cell
ME-MSC: Mouse Embryonic Mesenchymal Stem Cell
SHED-exos: Stem cells from Human Exfoliated Deciduous teeth-derived exosomes
ADSC-HExos: Adipose-Derived Stem Cell-derived Human Exosomes
pSGC: primary Submandibular Gland Cells
over-CaR-PSGs: Calcium-sensing Receptor-overexpressing Primary Salivary Gland cells
Treg: Regulatory T cell
Th17: T helper 17 cell
Apaf-1: Apoptotic protease activating factor 1
TNFR1: Tumor Necrosis Factor Receptor 1
DISC: Death-Inducing Signaling Complex
ER: Endoplasmic reticulum
GRP78: Glucose-Regulated Protein 78
PERK: Protein Kinase R-like ER Kinase
IRE1: Inositol-Requiring Enzyme 1
CHOP: C/EBP Homologous Protein
eIF2α: Eukaryotic Initiation Factor 2α
ATF4: Activating Transcription Factor 4
XBP1: X-Box Binding Protein 1
JNK: c-Jun N-terminal Kinase
